# Demand for cooling water reshapes global water-sustainable hydrogen production

**DOI:** 10.1038/s44458-026-00106-x

**Published:** 2026-06-26

**Authors:** Bernhard Wortmann, Daniel Arenas, Christoph Winkler, Jochen Linßen, Detlef Stolten, Heidi Heinrichs

**Affiliations:** 1 Forschungszentrum Jülich GmbH, Institute of Climate and Energy Systems, Jülich Systems Analysis (ICE-2), Jülich, Germany; 2 RWTH Aachen University, Chair for Fuel Cells, Faculty of Mechanical Engineering, Aachen, Germany; 3 University of Siegen, Chair for Energy Systems Analysis, Department of Mechanical Engineering, Siegen, Germany

**Keywords:** Environmental impact, Energy infrastructure

## Abstract

Green hydrogen is central to many decarbonization strategies, yet its water footprint is often reduced to the water consumed by electrolysis itself. As electrolyzer plants scale up, cooling can become a hidden water demand, especially in hot and water-stressed regions where many renewable hydrogen projects are planned. Here we combine a thermodynamic cooling model with climate reanalysis, global water-stress data and renewable capacity-factor maps to quantify evaporative-cooling water demand for electrolysis across regions and seasons. We show that cooling can dominate total water use and that high solar-resource regions frequently coincide with high water stress and high cooling demand. Wind-rich regions, in contrast, are more often located in cooler or more water-abundant settings. A composite Water Risk Index identifies where freshwater-based evaporative cooling is likely to require alternatives such as dry or hybrid cooling, desalination or reclaimed-water supply. Our results show that cooling technology and water sourcing are central to water-sustainable hydrogen planning.

## Abstract (web summary)

Renewable potential alone is insufficient to ensure water-sustainable hydrogen production, particularly for solar-powered electrolysis in arid and semi-arid regions, suggests a thermodynamic and spatial modeling study linking cooling demand, water scarcity and renewable capacity.

## Introduction

Hydrogen produced via water electrolysis powered by renewable energy is widely seen as a cornerstone of a decarbonized energy system^1,2^. Yet beyond the well-known electricity and material requirements, electrolysis also depends on a secure and sustainable water supply, a constraint that remains underestimated in global deployment strategies. While the stoichiometric water demand of electrolysis is modest, the cooling requirement can multiply total water use severalfold, particularly when evaporative systems are employed^3^. This challenge is most acute because large-scale hydrogen projects increasingly concentrate in water-stressed regions, such as the Middle East, Australia, and Chile^4^. In recent years global electrolyzer capacity has already doubled to 1.4 GW in 2023 and is expected to reach hundreds of gigawatts by 2030 and terawatt scale by mid-century^5–7^. Managing water sustainably will thus become a core prerequisite for the expansion of green hydrogen.

Although global water withdrawals for electrolysis would remain below one percent of freshwater supply^8^, local impacts can be substantial where freshwater is scarce. In such contexts, overlooking cooling water demand risks severely understating the true water footprint of hydrogen. Electrolysis requires roughly $9L/k{g}_{{H}_{2}}$ for the reaction itself, rising to around $10L/k{g}_{{H}_{2}}$ when purification losses are included^9–11^. In practice, however, thermal management dominates total water consumption as large electrolyzer stacks generate substantial waste heat that must be removed to maintain efficiency and durability^12,13^.

Different cooling technologies offer distinct trade-offs between water use, efficiency, and cost. Once-through systems withdraw large volumes but return most water, resulting in limited net consumption where regulation allows^14^. Evaporative cooling withdraws less but incurs substantial consumptive losses through evaporation, remaining attractive for its simplicity and performance^3^. Dry (air) cooling eliminates water use but can have up to eight times higher capital costs than evaporative cooling and reduces efficiency, especially in hot climates^3,15^. Choosing the appropriate cooling system is therefore not a peripheral engineering detail but rather a strategic determinant of hydrogen’s geographic and economic feasibility^16–18^.

**Fig. 1:**
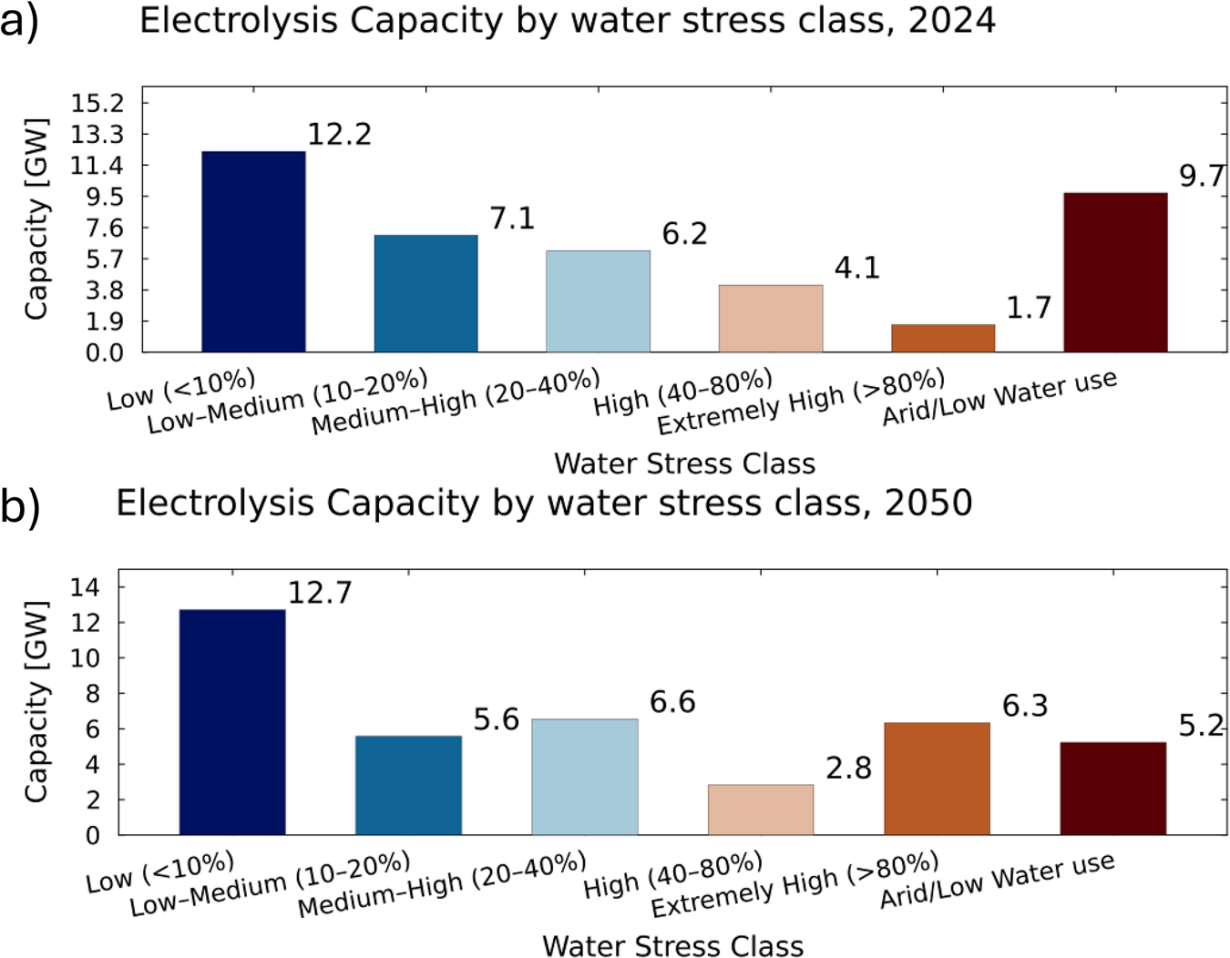
PEM and alkaline electrolysis capacity distribution by water-stress class. **a** Distribution of announced proton exchange membrane and alkaline electrolysis project capacity using 2024 water-stress classes. **b** Distribution of the same project capacity using 2050 water-stress projections under a business-as-usual scenario. Bars show total electrolysis capacity in gigawatts across Aqueduct water-stress categories. Water-stress data are from Aqueduct 4.0, and project-capacity data are from the International Energy Agency Hydrogen Projects Database^19,29^.

Strikingly, nearly half of all announced large-scale electrolysis projects are situated in arid or water-stressed regions (Fig. 1a), a share projected to grow under future climate scenarios (Fig. 1b)^19^. In the Aqueduct framework, water stress, defined as withdrawals exceeding 40 % of renewable supply, already signals competition for local water sources among users^20^. Deploying evaporative cooling on a large scale in such regions could therefore intensify conflicts with agriculture and other water-intensive industries^21^, an effect further exacerbated by high ambient temperatures that raise cooling demand precisely where water is scarcest^12,22^.

Despite these challenges, global assessments of hydrogen’s water footprint have so far focused almost exclusively on reaction water, typically citing values of $10L/k{g}_{{H}_{2}}$^23,24^. For example, Hydrogen Europe estimates that producing $10M{t}_{{H}_{2}}{y}^{-1}$ would use just 0.005 % of EU freshwater, ignoring cooling and additional treatment, as well as local freshwater competition ^25^. A few recent studies incorporate local water availability^10,26^, but their scope remains regional. Only Ellersdorfer et al. have explicitly quantified cooling water demand^12,15^, yet without linking it to global climate, water-stress or capacity-factor patterns, lacking a global spatially resolved contextualization. This gap is practically relevant, as recent feasibility studies in water-constrained regions, such as Australia, already report consideration of water-saving cooling configurations, including adiabatic cooling, in response to local water constraints^27^. However, such decisions remain project-specific and lack a systematic global quantitative basis for identifying where such alternatives are required.

Here, we address this gap through a global, seasonally and spatially resolved assessment of water consumption for proton exchange membrane (PEM) and alkaline (AEL) electrolysis, with a particular focus on evaporative cooling. We couple a thermodynamic cooling model with ERA5 climate data^28^ and Aqueduct water-stress metrics^29^ to quantify how local meteorology and water availability shape cooling-water demand. Using principal-component analysis, we construct a composite water-risk index that integrates physical water consumption and resource scarcity. Finally, we compare these risk patterns with global photovoltaic (PV) and onshore wind capacity factors to identify where renewable potential aligns or conflicts with water suitability for hydrogen production. This integrated framework provides technology-specific siting guidance and highlights where alternative cooling strategies or non-freshwater sources will be essential for scaling the hydrogen economy sustainably.

## Results

### Cooling water demand varies strongly with climate and season

Cooling water demand for electrolysis depends strongly on local climate conditions and varies substantially over the year, as evaporative cooling is sensitive to temperature and humidity. Seasonal fluctuations can therefore amplify or relieve cooling-water requirements relative to annual averages, even in regions with moderate mean conditions. Accounting for this temporal variability is important for interpreting modeled water demands and their implications for hydrogen production. We therefore analyze seasonal patterns in cooling-water demand across global climates and quantify how these patterns modify the spatial distribution of water requirements.

**Fig. 2:**
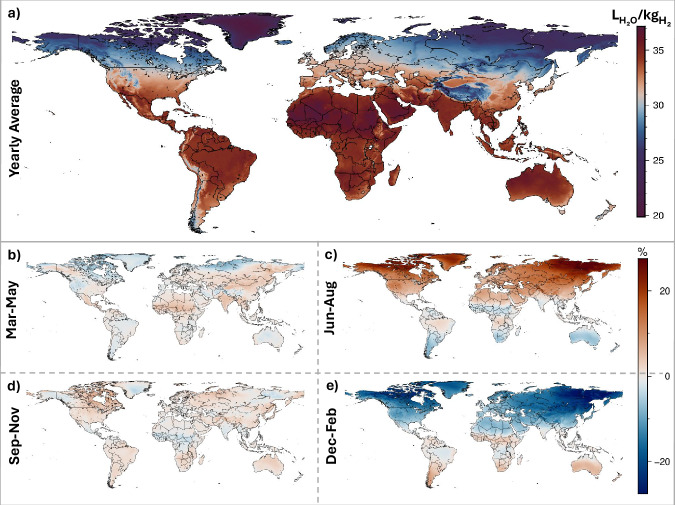
Water consumption of electrolysis using evaporative cooling. **a** Annual average specific water consumption of electrolysis using evaporative cooling, calculated from long-term mean ambient and dew-point temperatures from ERA5 reanalysis data. **b–e** Seasonal deviations from the annual average for March--May, June--August, September--November, and December--February, respectively. **a** shows total specific water consumption in litres of water per kilogram of hydrogen, including reaction water and cooling-water demand. **b–e** show percentage deviations from the annual average. Blue shading indicates lower values or negative deviations, and orange-to-red shading indicates higher values or positive deviations^28^.

Figure 2a shows the modeled specific water consumption of electrolysis using evaporative cooling, expressed in ${L}_{{{{\rm{H}}}}_{2}{\rm{O}}},{{{\rm{kg}}}}_{{{{\rm{H}}}}_{2}}^{-1}$, and including both reaction water and cooling demand. Values are derived from long-term annual averages of ambient and dew-point temperatures from ERA5 reanalysis data^28^.

Seasonal deviations from this annual baseline are illustrated in Fig. 2b–e showing the percentage change in specific water consumption during each three-month period. These maps reveal that temperature and humidity fluctuations modulate cooling-water demand throughout the year and reveal the seasonal amplification or relief of evaporative load across different climate zones.

Distinct geographical patterns emerge. The lowest specific average water consumption, around 19 ${L}_{{{{\rm{H}}}}_{2}{\rm{O}}},{{{\rm{kg}}}}_{{{{\rm{H}}}}_{2}}^{-1}$, occurs in high-latitude and colder regions. The highest values, reaching up to 39 ${L}_{{{{\rm{H}}}}_{2}{\rm{O}}},{{{\rm{kg}}}}_{{{{\rm{H}}}}_{2}}^{-1}$, are concentrated in hot and arid areas, such as Northern Africa, the Middle East and North Africa (MENA), inland Australia, parts of South Asia, and the U.S. Southwest. Coastal and temperate regions generally exhibit moderate annual consumption (Fig. 2a). Seasonal deviations are relatively small (within  ± 7%) during March-May (Fig. 2b) and September-November (Fig. 2d). By contrast, deviations intensify during June-August and December-February (Fig. 2c, e), reaching up to  ± 25% in many regions. These peaks reflect the amplifying effects of high temperatures and low relative humidity on evaporative cooling demand, and unveil otherwise overlooked water-intensive regions.

**Fig. 3:**
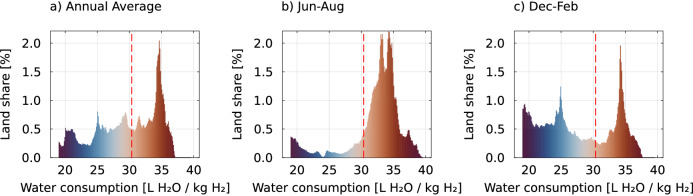
Global distribution of cooling-water consumption for electrolysis using evaporative cooling. **a**Distribution of annual average specific water consumption across global land areas. **b**Distribution for June--August. **c**Distribution for December--February. Histogram bars show the share of global land area in each specific water-consumption bin. Bar colors follow the same blue-to-red water-consumption scale as Fig. 2aand are fixed to the annual scale for comparability. Vertical red dashed lines indicate the median value of the annual average distribution.

To complement the spatial and seasonal analysis, Fig. 3 examines the global distribution of specific water consumption values, highlighting both typical and extreme conditions. The distributions confirm that a substantial share of global land lies above the statistical median of 30 ${L}_{{{{\rm{H}}}}_{2}{\rm{O}}},{{{\rm{kg}}}}_{{{{\rm{H}}}}_{2}}^{-1}$, indicating pronounced seasonal shifts. For the annually averaged ERA5 data (Fig. 3a), 50% of global land exhibits water requirements above the global median. Marked seasonal shifts are evident. During June-August (Fig. 3b), 83% of land lies above the annual median, while during December-February (Fig. 3c) this share falls to 36%. This pronounced swing illustrates how peak-season climatic conditions can push otherwise moderate locations into higher water-consumption regimes, potentially challenging the sustainability of evaporative cooling depending on local water availability.

Pronounced seasonal increases in cooling-water demand occur across mid-latitude continental interiors in the Northern Hemisphere, notably the U.S. interior and Southwest, southern Europe and the Mediterranean rim, and Central Asia/North China, where June-August deviations commonly reach  + (15 − 25%) relative to the annual baseline. In contrast, persistently hot-arid regions (e.g., the central Middle East) show high absolute consumption but smaller relative swings, while maritime/coastal zones exhibit damped variability. During December-February, the same interiors display  − (10−20%) reductions relative to annual means. These patterns imply that many otherwise moderate sites could be seasonally pushed above design-relevant ranges in summer, affecting cooling system selection (evaporative vs. hybrid/dry), seasonal water allocation and permitting, and operational planning (e.g., maintenance in low-demand months, load shifting/curtailment across peak heat).

**Fig. 4:**
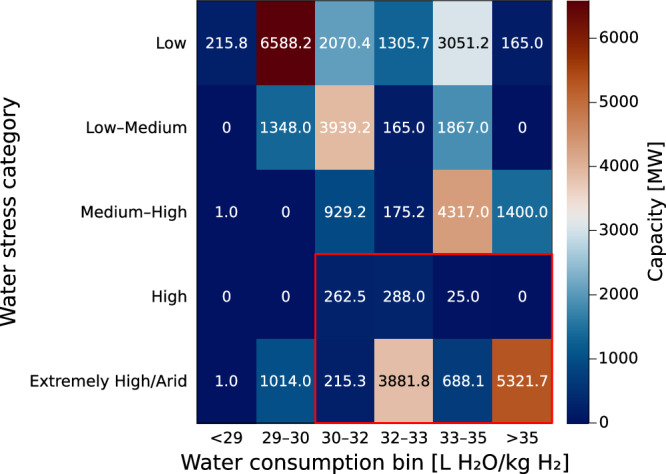
Electrolysis capacity distribution by water-stress category and evaporative-cooling water demand. Cell colors indicate total announced electrolysis capacity in megawatts. The x-axis shows bins of modeled specific water consumption for electrolysis using evaporative cooling, and the y-axis shows Aqueduct water-stress categories. The red box highlights project capacities located in water-stressed regions with specific water requirements above the global median value.

To illustrate the practical relevance of these seasonal water-demand patterns we allocate the announced PEM and AEL project capacities^19^ to water stress categories^29^ and modeled water consumption bins for evaporative cooling (Fig. 4). The resulting distribution reveals that a substantial share of planned capacity (around 25%, 10.68 GW) is located in regions where water requirements are high and local water resources are under moderate to severe stress. This concentration highlights the importance of jointly considering cooling water demand and water availability when assessing the suitability of cooling technologies at prospective hydrogen production sites.

### A composite index reveals hotspots of water risk

**Fig. 5:**
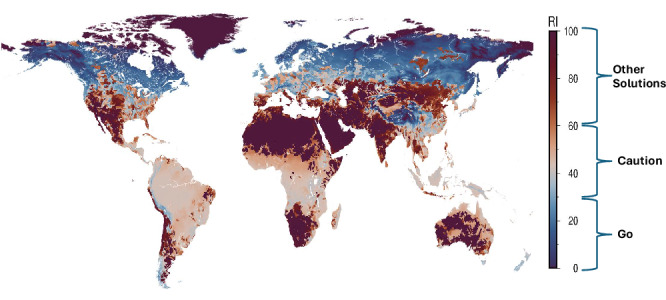
Global Water Risk Index for hydrogen production using evaporative cooling. The composite Water Risk Index is scaled from 0 to 100 and combines modeled evaporative-cooling water consumption with regional water-stress levels. Colors denote risk categories for local water resources: Go, 0–30, low risk; Caution, 30–60, potential risk; and Other Solutions, 60–100, high risk.

To quantify the combined exposure of water electrolysis to water scarcity and evaporative cooling demand, we develop a composite Water Risk Index (RI; see Section 4.3). The index integrates modeled evaporative-cooling water consumption (Section 2) with the spatially resolved Aqueduct 4.0 water-stress metric from the year 2023^29^. Expressed on a normalized 0-100 scale, the RI represents the potential vulnerability of evaporatively cooled electrolysis to local water-supply bottlenecks. Fig. 5 illustrates the global distribution of the RI, with dark blue areas denoting low risk and dark red areas indicating high risk. For interpretability, the RI is grouped into three categories: Go (RI = 0–30), where evaporative cooling is unlikely to exert notable pressure on water resources; Caution (RI = 30–60), where local impacts may arise; and Other Solutions (RI = 60–100), where such cooling would almost certainly intensify water scarcity if alternatives are not deployed (see Section 4.3).

The global distribution of the RI exhibits a distinct climatic gradient, with low-risk values dominating in humid, high-latitude regions and pronounced hotspots concentrated in arid and semi-arid zones, including North Africa, MENA, Central Asia, India, and the southwestern United States. Overall, 39.7% of the global land surface falls within Go areas, 32.3% within Caution, and 27.9% within Other Solutions zones.

Regions characterized by groundwater depletion combined with high temperatures, low humidity, and intensive water withdrawals, such as the North China Plain, display the expected elevated RI values, consistent with previous findings^30,31^. Moderate risk levels classified as Caution occur in countries, such as Brazil, which historically experience low overall water stress but show comparatively high evaporative-cooling demand and have experienced increasing drought frequency in recent years^32^. Beyond these broad climatic patterns, several regions deviate from the expected trend, exhibiting either unusually high or unexpectedly low risk levels. Cases with unexpectedly low RI values appear in the Democratic Republic of the Congo, where high modeled water consumption is offset by exceptionally abundant freshwater resources that maintain low stress levels^33,34^. Conversely, parts of Southern Europe, particularly the Po Valley in northern Italy, exhibit higher-than-anticipated RI values, largely driven by recurrent summer droughts and heat extremes that exacerbate local water scarcity^35,36^.

**Fig. 6:**
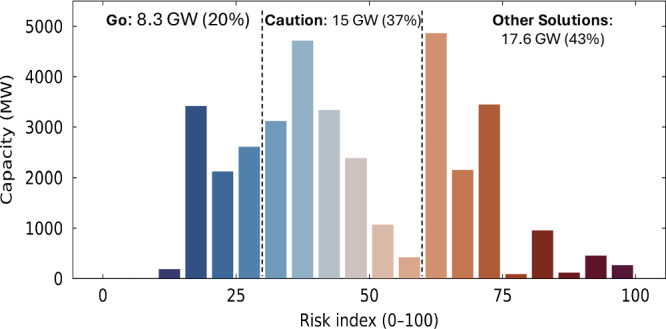
Distribution of installed and planned electrolysis capacity across Water Risk Index classes. Bars show total proton exchange membrane and alkaline electrolysis project capacity in megawatts within each Water Risk Index interval. Dashed vertical lines indicate the thresholds separating Go, 0–30; Caution, 30–60; and Other Solutions, 60–100. Text annotations above the plot show the corresponding shares of global project capacity in each class: 20%, 37%, and 43%, respectively.

Although the majority of global land area falls within the Go category, the distribution of planned electrolysis capacity tells a different story (Fig. 6). Only about 20% of PEM and AEL projects are located in Go regions, while 37% fall within Caution and 43% within Other Solutions zones. This apparent imbalance reflects the tendency of hydrogen facilities to cluster in industrial hubs, often characterized by elevated water stress, or in regions with favorable renewable-energy capacity factors, which frequently coincide with higher aridity and temperatures. To further examine these interactions, the following section contextualizes the water-risk index with renewable-energy capacity factors to assess the spatial exposure of hydrogen production to cooling-water constraints.

### Renewable potential often coincides with high water risk

**Fig. 7:**
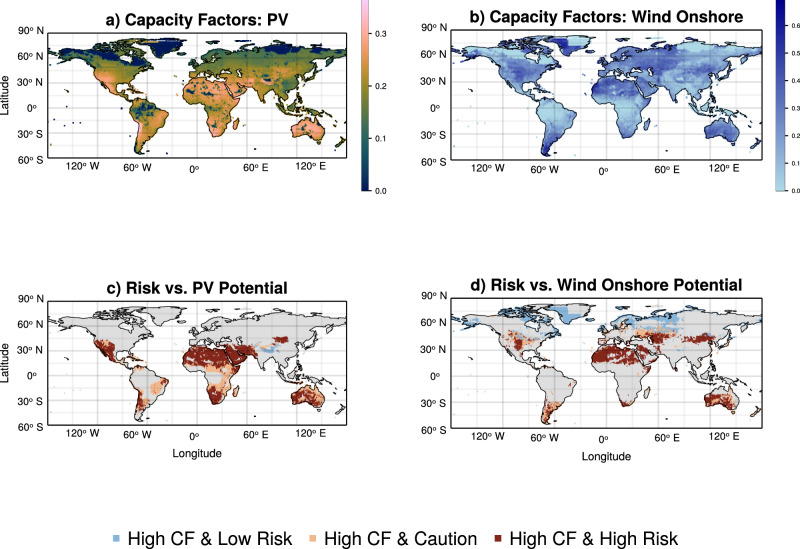
Photovoltaic and onshore wind capacity factors with Water Risk Index overlay. **a** Global photovoltaic capacity factors. **b** Global onshore wind capacity factors. **c**, Locations in the top quartile of photovoltaic capacity factors, classified by Water Risk Index category. **d** Locations in the top quartile of onshore wind capacity factors, classified by Water Risk Index category. Blue points indicate high-capacity-factor locations in the Go class, orange points indicate high-capacity-factor locations in the Caution class, and red points indicate high-capacity-factor locations in the Other Solutions class. Gray land areas indicate locations outside the top-quartile subset of capacity factors. CF denotes capacity factor.

We translate the composite water-risk index for evaporative cooling into a concrete siting signal for green-hydrogen projects by overlaying it with global capacity-factor maps for PV and onshore wind (Fig. 7a, b)^37,38^. Specifically, we flag grid cells in the top quartile of capacity factors (high CF) and classify them by water risk into Go, Caution, and Other Solutions zones (Fig. 7c, d). This approach turns the index into actionable guidance quantifying where abundant renewable resources align with, or conflict with, freshwater suitability for evaporative cooling, and allows a technology-specific comparison between PV and wind.

Across both technologies, regions of high renewable potential tend to coincide with arid and semi-arid climates that are also characterized by structural water stress. Strong solar irradiance and stable wind regimes in these zones arise under conditions of low humidity and scarce precipitation, the same climatic drivers that intensify evaporative water losses and limit renewable freshwater availability. As a result, the global pattern reveals a pronounced overlap between high renewable potential and high water risk, forming contiguous belts of high-potential / high-risk zones across subtropical latitudes, extending from the western United States through MENA, southern Africa, Chile’s Atacama Desert, and Australia’s interior, while temperate and high-latitude regions remain comparatively low-risk and water-abundant.

This coupling is particularly evident in photovoltaic (PV) resources, where solar abundance and water scarcity are most closely linked. Globally, 63.3% of high-PV capacity-factor locations fall within Other Solutions zones for evaporative cooling, 34.2% within Caution zones, and only 2.5% within Go zones. The highest-risk overlaps are concentrated across the MENA region, the western United States, Chile’s Atacama Desert, southern Africa, and Australia’s interior, all areas that combine high irradiance with chronic water stress. From a technical perspective, these results confirm that large-scale deployment of evaporative cooling systems in PV-dominated hydrogen projects would be infeasible without substantially increasing the withdrawal of freshwater or deploying seawater desalination. Thus, in the majority of high-solar regions, water availability and not land or sunlight, emerges as the primary limiting factor for siting electrolyzer capacity. At a system level, this conflict between renewable abundance and water scarcity already manifests in project planning. Flagship ventures, such as the NEOM Green Hydrogen Project in Saudi Arabia (2.2 GW)^39^, the Hyphen Hydrogen Energy Project in Namibia (3 GW)^40^, and the Pilbara Green Hydrogen Project in Australia^41^ are all located in high-risk zones. Projects of this scale are therefore unlikely to rely on freshwater supplies when adopting evaporative cooling. Instead, they must integrate alternative cooling pathways, dry or hybrid configurations, or couple electrolysis with seawater desalination and waste-heat recovery, as recently proposed by Ellersdorfer et al^12,15^.

The picture changes when overlaying the water-risk index with onshore wind capacity factors (Fig. 7d), revealing a markedly less severe coupling between renewable potential and water scarcity. Globally, 43.9% of high-wind regions fall within Other Solutions zones, 25.1% within Caution, and 31.0% within Go areas, indicating that a substantial portion of prime wind locations lie in water-sustainable environments. High-risk overlaps occur in the familiar arid belts of MENA and Australia, yet the majority of wind-rich regions, particularly in temperate and coastal zones, remain largely unconstrained by freshwater limitations. From a technical standpoint, this weaker correlation reflects the distinct climatic drivers of wind energy. Strong and persistent wind regimes often arise along coastal, mountainous, or high-latitude corridors, where ambient temperatures are lower and relative humidity is higher. Consequently, electrolysis systems powered primarily by wind are less exposed to operational or environmental risks associated with evaporative cooling, enabling a broader geographic portfolio of feasible hydrogen production sites. At a system level, this decoupling implies that wind-dominated hydrogen pathways can act as a natural hedge against climate-driven water scarcity, complementing solar-based production in arid zones. However, localized challenges remain where infrastructure or freshwater accessibility, rather than climatic aridity, is the limiting factor. A key example is Patagonia, a globally recognized wind hotspot and emerging hydrogen-export region. Despite its relatively humid climate, parts of southern Patagonia experience water stress, increasing water risks. Consequently, large-scale initiatives, such as the Magallanes Hydrogen Project plan to rely on seawater desalination for process water^42^, a strategy likely required across the wider Patagonian corridor. In contrast, northern Europe, Russia, and Canada combine high wind potential with low water risk and robust water systems, indicating that large-scale electrolysis there could proceed with minimal freshwater constraints, regardless of cooling technology.

Taken together, the contrasting spatial patterns of PV and wind highlight that water risk is highly technology-specific. Solar-driven hydrogen production is concentrated in arid, water-stressed regions, whereas wind resources are more spatially decoupled from freshwater scarcity. This divergence underscores the need for region- and technology-tailored cooling and water-sourcing strategies, which are central considerations for sustainable hydrogen deployment in the following discussion.

## Discussion

Cooling-water demand is an important yet often underappreciated dimension of the water sustainability of green hydrogen production. Our analysis indicates that under evaporative cooling, water use is largely driven by thermal management requirements and varies strongly with local climate conditions and seasonality. As a result, assessments based solely on stoichiometric water demand or annual-average conditions risk underestimating water constraints at prospective hydrogen production sites.

By integrating seasonally resolved cooling-water demand with local water availability, this work highlights how water constraints reshape the global geography of hydrogen deployment. The resulting patterns indicate that renewable potential alone is insufficient to ensure water-sustainable hydrogen production, particularly for solar-powered electrolysis in arid and semi-arid regions. Instead, the choice of cooling technology and water sourcing emerges as a decisive factor alongside energy yield.

The results demonstrate that the choice of cooling is a decisive determinant of electrolysis feasibility, rather than a secondary design parameter. Evaporative systems multiply total water use severalfold (factor of 2-5) beyond the stoichiometric requirement, meaning that water demand is dominated by thermal management rather than the electrochemical reaction itself. When combined with renewable-energy patterns, this constraint becomes particularly pronounced for solar-powered electrolysis, where more than sixty percent of the world’s high-irradiance, high-capacity-factor regions coincide with Other Solutions water-risk zones. In these environments, freshwater scarcity, not land or solar resources, emerges as the primary limiting factor for hydrogen production. By contrast, wind-driven electrolysis shows a much weaker coupling to water risk, as favorable wind regimes often occur in temperate, coastal, or high-latitude areas with comparatively abundant water resources. This divergence implies a geographical and climatic complementarity between solar and wind hydrogen pathways: while solar projects in arid zones will require dry or hybrid cooling and likely rely on non-freshwater sources, humid mid-latitudes and coastal regions remain viable for conventional evaporative systems.

Beyond the spatial analysis, our findings carry direct techno-economic and system-level implications for large-scale hydrogen deployment. The choice of cooling technology strongly shapes both capital and operational costs, process efficiency, and permitting feasibility. Recent techno-economic assessments indicate that evaporative cooling can be up to eight times cheaper to implement than dry cooling while maintaining higher thermal efficiency under most operating conditions^15^. However, this cost advantage comes at the expense of substantial freshwater consumption, implying that economic attractiveness and water sustainability diverge in arid regions. For coastal hydrogen hubs in the Middle East, Australia, or Chile, this tension makes coupling with desalination facilities inevitable. While desalinated water is technically viable with low impact on the overall electrolysis plant CAPEX^43^, a first-order estimate based on the cooling-water volumes derived in this study indicates that supplying the additional 20–40 L kg^−1^ H_2_ of cooling water via seawater reverse osmosis would increase electricity demand by only about 0.08–0.25 kWh kg^−1^ H_2_, corresponding to roughly 0.15–0.50% of system-level electrolysis energy, while adding around 0.02–0.09 USD kg^−1^ H_2_, equivalent to about 0.3–4.5% of cost optimized levelized cost of hydrogen (LCOH) projected by the IEA for 2030 (see Supplementary Note 2)^4^. Its associated energy and brine-management burdens must be factored into overall hydrogen economics and environmental assessments^15^. Even though desalination offers a promising mitigation pathway at relatively low cost, brine management introduces additional environmental and operational burdens that are often overlooked in techno-economic assessments. Disposal of concentrated brine can elevate salinity and chemical loads in coastal ecosystems, requiring energy-intensive dilution or specialized treatment processes that add 0.3–0.7 USD m^−3^ to the cost of water production^44–46^. Compared with reaction-water-only desalination assessments, including cooling demand increases the associated energy and cost penalty by roughly a factor of three to five^11,47^. These hidden costs and externalities may, in some contexts, create the wrong incentives to rely on scarce freshwater resources rather than to invest in more sustainable non-freshwater supply and brine-management solutions. Consequently, integrating cooling-water and desalination requirements into techno-economic and life-cycle analyses is essential to avoid underestimating total water and energy use, especially in PV-driven electrolysis. Another relevant mitigation pathway is adiabatic cooling, which occupies an intermediate position between evaporative and fully dry systems. In such configurations, heat is removed primarily through dry cooling, while water use is limited to above-ambient thresholds or during peak thermal conditions. This can reduce aggregate water demand relative to conventional evaporative towers while avoiding some of the full efficiency and cost penalties associated with entirely dry cooling. Recent Australian feasibility studies already point in this direction: for example, the ARENA-supported ATCO ScaleH2 study identified adiabatic cooling as the preferred option for the assessed site conditions, highlighting that water-saving cooling strategies are already being considered in practice in response to the type of regional water-risk patterns identified here^27^.

Furthermore, the observed seasonal variability of up to  ± 25% in cooling water demand introduces an additional operational dimension. In regions with pronounced climatic oscillations, such as the Mediterranean Basin and inland continental climates, peak summer conditions can temporarily elevate cooling demand beyond design capacities, constraining electrolysis operation. Seasonal load-shifting or hybrid cooling systems could mitigate peaks, but require explicit planning. Collectively, these considerations underscore that water-cooling design choices are not isolated engineering details but central determinants of the spatial, economic, and environmental feasibility of the global hydrogen economy.

The broader policy relevance of these findings lies in integrating water considerations into the governance frameworks shaping the hydrogen economy. To date, most certification and financing schemes for green hydrogen focus almost exclusively on carbon intensity, narrowly crafted around technical issues, including water consumption^48^. Our results indicate that water availability should become a formal sustainability criterion in project approval, financing, and certification. The global water-risk maps and composite index presented here provide a practical screening tool for policymakers, investors, and developers to pre-assess project feasibility, identify Other Solutions regions, and mandate mitigation measures, such as non-freshwater sourcing or hybrid cooling. Incorporating water-energy-climate interactions into national hydrogen road maps would help prevent emerging competition between hydrogen production, agriculture, and domestic water use. By adopting such integrated planning approaches, governments and financial institutions can steer investment toward locations and technologies that ensure both climate and resource sustainability.

While this framework abstracts from short-term operational dynamics and transient climatic extremes, it captures the dominant drivers of cooling-water demand at spatial and seasonal scales relevant for strategic planning. Accordingly, short-duration heatwave or drought events within a season are not explicitly resolved and may temporarily increase cooling-water demand beyond the climatological seasonal averages reported here; these effects should be addressed in site-specific design and operational assessments. Likewise, adiabatic cooling is not explicitly represented in the present framework, since its performance depends on site- and design-specific operating thresholds and ambient conditions that are not readily transferable to a globally harmonized screening model. The findings highlight that the choice of cooling technology and water sourcing is not a secondary design consideration but a central determinant of water-sustainable hydrogen deployment.

## Methods

### Evaporative-cooling model

Cooling water demand in evaporative towers is driven by the heat rejected from the electrolyzer stack and the ability of ambient air to absorb this heat as moisture^46^. The rejected heat ${\dot Q}_{loss}$ depends on electrolyzer efficiency:

1 $${\dot {Q}}_{loss} = {\dot {m}}_{{H}_{2}}\cdot \left(\frac{1-{\eta }_{el}}{{\eta }_{el}}\right)\cdot HHV$$

where ${\dot {m}}_{{H}_{2}}$ is the hydrogen mass flow, *η*_*el*_ the electrical efficiency typically ranging around 65%^12,15^, and *HHV* the higher heating value of hydrogen^49^. The model is formulated at the cooling tower level: evaporative water demand is driven by the total stack heat load ${\dot {Q}}_{{{\rm{loss}}}}$ and ambient psychrometric conditions, which together set the amount of water that must be evaporated to reject waste heat to the atmosphere. Because commercial PEM and alkaline electrolyzers (AEL) operate in largely overlapping temperature envelopes (PEM at 50 °C–80 °C^50,51^ and commercial AEL at 60 °C–90 °C^50–52^), the efficiency-driven heat balance in Eq. (1) is formulated as technology-agnostic at this level of abstraction. This is a deliberate scope choice consistent with the joint capacity treatment of PEM and AEL throughout this work and with the resolution of the global ERA5-driven analysis.

The total make-up flow rate of cooling water is

2 $${\dot {m}}_{makeup}={\dot {m}}_{evap}+{\dot {m}}_{drift}+{\dot {m}}_{bd}.$$

where the three terms represent evaporative, drift, and blow-down losses, respectively. Evaporative loss dominates and scales linearly with ${\dot{Q}}_{{{\rm{loss}}}}$ (equation (3)), modulated by local dry-bulb temperature, relative humidity, and wet-bulb approach^53^. Drift and blow-down are minor additions, typically *d* = 0.0005–0.2% and *n* = 3 cycles of concentration^53,54^.

3 $${\dot {m}}_{evap}\propto {\dot {Q}}_{loss},$$

Full thermodynamic relations and derivations are given in Supplementary Method 1. Sensitivity analyses of input parameters are provided in Supplementary Note 1. ERA5 reanalysis data (2010–2020, 0.25^∘^ resolution)^28^ provide dry- and dew-point temperatures used to estimate regional evaporation potential. All parameter ranges and sources are summarized in Table 1.

**Table 1:** Key model parameters and constants used in the evaporative-cooling model

Parameter	Symbol	Value / Range	Unit	Source
Electrolyzer efficiency	*η*_el_	0.65	–	^12,15^
Higher heating value of hydrogen	HHV	141.8	MJ kg^−1^	^60^
Drift loss fraction	*d*	0.0005–0.002	–	^54^
Cycles of concentration	*n*	3	–	^54^
Air–water approach	Δ*T*_app_	3–5	^∘^C	^61,62^
Ambient temperature	*T*_air_	ERA5 mean	^∘^C	^28^
Dew-point temperature	*T*_dew_	ERA5 mean	^∘^C	^28^
ERA5 coverage	–	2010–2020	–	10-year climatology
ERA5 resolution	–	0.25^∘^	–	Reanalysis grid
Water-stress dataset	–	Aqueduct 4.0	–	^29^

### Water-stress data

Regional water availability is characterized using the World Resources Institute (WRI) Aqueduct 4.0 Water-Risk Atlas^29^ from the year 2023. Water stress is defined as the ratio of annual water withdrawals to the renewable surface and groundwater supply. Aqueduct classifies stress as low ( < 10%), low-medium (10–20%), medium-high (20–40%), high (40–80%), and extremely high ( > 80 %). Water stress values above 40% indicate high competition for local water resources^29^. Regions displaying these values are therefore referred to as water-stressed. The dataset provides global coverage at 10 × 10 km resolution, resampled to the ERA5 grid for consistency. Linking these stress levels with modeled cooling-water demand enables identification of regions where electrolysis may face competition for freshwater.

### Risk index construction

To evaluate the combined impact of water consumption and water stress, we define a composite Water Risk Index *Z*_*i*,*s*_ for each location *i* and season *s*:

4 $${Z}_{i,s}={\alpha }_{s}{x}_{i,s}+{\beta }_{s}{y}_{i,s},$$

where *x*_*i*,*s*_ is the modeled cooling water consumption (Section 4.1), and *y*_*i*,*s*_ is the WRI Aqueduct water stress value^29^. Both indicators are standardized and expressed as standard deviations to ensure comparability across regions and seasons.

The weights (*α*_*s*_, *β*_*s*_) determine the relative contribution of each parameter. Rather than assigning them arbitrarily, we derive the weights using principal component analysis (PCA)^55,56^. PCA identifies the linear combination of *x*_*i*,*s*_ and *y*_*i*,*s*_ that captures the largest share of variance across seasons, with the component loadings indicating the relative influence of each variable. This procedure is performed separately for each season to capture seasonal differences in the relative importance of water consumption and water stress. The detailed derivation of the covariance matrix and eigenvector decomposition is provided in the Supplementary Information.

Seasonal risk scores *Z*_*i*,*s*_ are aggregated into an annual index by applying seasonal weights *ω*_*s*_:

5 $${I}_{i}={\sum }_{s}{\omega }_{s}{Z}_{i,s},\,\sum {\omega }_{s}=1,$$

and rescaled to the interval [0, 100] for interpretation. Sites are then categorized as Go ($\in [0,30]$), Caution ($\in [30,60]$), or Other Solutions ($\in [60,100]$), where such cooling would almost certainly intensify water scarcity if alternatives, such as dry cooling, adiabatic/hybrid cooling, desalination, or reclaimed-water supply, are not deployed. Thresholds correspond approximately to the terciles of the global distribution; shifting cutoffs by  ± 5 points alters global area shares by  < 5%, confirming robustness. The RI is intentionally formulated as a baseline screening metric for freshwater-based evaporative cooling; desalination and reclaimed-water supply are therefore treated as mitigation options outside the core index rather than embedded assumptions.

### Renewable capacity factors

Renewable electricity capacity factors used in this study are based on the method from Winkler et al. ^37^ and Ishmam et al^38^. The data was generated in cooperation with the International Energy Agency (IEA) and is taken from the Global Hydrogen Review^4^. The data cover photovoltaic and onshore wind generation potentials from the year 2018, derived from ERA5 reanalysis in combination with the Global Solar and Wind Atlases, using the ETHOS.RESKIT model^57,58^ on previously filtered, land-eligible areas.

Although not directly used in the model calculations, these capacity factors provide important context for interpreting the composite water risk indices. Regions with high renewable potential are typically seen as prime candidates for green hydrogen projects, yet they often coincide with hot or arid climates where evaporative cooling demands are highest. Linking water risk with renewable capacity factors, therefore, allows us to highlight where promising hydrogen production sites may face critical water constraints and where alternative cooling strategies may be required.

## Supplementary information

Transparent Peer Review file

Supplementary Information Demand for cooling water reshapes global water-sustainable hydrogen production

## Data Availability

The processed data and figure-source data needed to reproduce the figures, supplementary analyses, and headline numerical results reported in this study will be deposited in a public Zenodo repository before publication: 10.5281/zenodo.20340784^59^. The repository includes processed cooling-water-consumption outputs, Water Risk Index outputs, project-capacity overlays, sensitivity-analysis data, reference figures, and metadata describing all external input datasets. Raw third-party datasets are not redistributed where they are publicly available from the original providers, large in size, or subject to provider-specific access conditions. ERA5 climate data are available from the Copernicus Climate Data Store. Aqueduct 4.0 water-stress data are available from the World Resources Institute. Hydrogen-project data are available from the International Energy Agency. Renewable capacity-factor input data and all further external datasets are documented in the repository metadata and cited in the manuscript.

## Code availability

The custom code used to process the input datasets, calculate evaporative-cooling water demand, construct the Water Risk Index, perform sensitivity analyses, and generate figure-source data will be deposited in the same Zenodo repository before publication: 10.5281/zenodo.20340784^59^. The repository contains a frozen publication-specific code snapshot, documentation of the processed-data reproduction workflow, and software environment information. The code is provided to reproduce the analyses reported in this article and does not include unrelated exploratory scripts or unpublished follow-up work.
