# Supplementary material for: Demand for cooling water reshapes global water-sustainable hydrogen production
Source: Commun Sustain. 2026 Jun 26;1(1):103. doi: 10.1038/s44458-026-00106-x (PMC13303071; doi:10.1038/s44458-026-00106-x)
Supplement: Supplementary file 2 — Supplementary Information Demand for cooling water reshapes global water-sustainable hydrogen production [file 44458_2026_106_MOESM2_ESM.pdf]

Supplementary Information

Demand for cooling water reshapes global water-sustainable hydrogen production

Bernhard Wortmann[1,2], Daniel Arenas[2], Christoph Winkler[1,2], Jochen Linßen[1], Detlef Stolten[1,2], Heidi Heinrichs[1,3]

[1]Forschungszentrum Jülich GmbH, Institute of Climate and Energy Systems, Jülich Systems Analysis (ICE-2), 52425 Jülich, Germany
[2]RWTH Aachen University, Chair for Fuel Cells, Faculty of Mechanical Engineering, 52062 Aachen, Germany
[3]University of Siegen, Chair for Energy Systems Analysis, Department of Mechanical Engineering, 57076 Siegen, Germany

Supplementary Methods

Supplementary Method 1: Thermodynamic cooling-water model

The cooling water consumption model is built on fundamental thermodynamic relationships while deliberately avoiding excessive technological detail. The starting point for estimating evaporative water losses is the heat rejected by the electrolyzer stack, which is a function of its electrical efficiency:

$$\dot{Q}_{loss} = \dot{m}_{H_2} \cdot \left(\frac{1 - \eta_{el}}{\eta_{el}} \right) \cdot HHV \tag{1}$$

where $\dot{m}_{H_2}$ is the hydrogen mass flow rate, η_{el} is the electric efficiency and HHV the water electrolyzer higher heating value. The total water consumption of an evaporative cooling tower is represented by the required makeup water rate $\dot{m}_{makeup}$ (equation 2) which accounts for all water losses during operation. These losses include evaporative losses $\dot{m}_{evap}$, which are directly related to the heat rejection $\dot{Q}_{loss}$, as well as drift losses $\dot{m}_{drift}$ and the blow-down losses $\dot{m}_{bd}$. Drift losses refer to small liquid water droplets that are entrained in the exhaust air stream and carried out of the cooling tower during operation. Blow-down losses arise from the periodic discharge of circulating cooling water to control the accumulation of dissolved salts and impurities, which would otherwise degrade heat transfer performance [1].

$$\dot{m}_{makeup} = \dot{m}_{evap} + \dot{m}_{drift} + \dot{m}_{bd} \tag{2}$$

The evaporative water loss rate $\dot{m}_{evap}$ is calculated from the change in specific humidity of the cooling air as it passes through the tower [1, 2].

$$\dot{m}_{evap} = \dot{m}_{\alpha} \cdot (\omega_{\alpha 2} - \omega_{\alpha 1}) \tag{3}$$

Here, $\dot{m}_{\alpha}$ denotes the mass flow rate of air circulating through the cooling tower, while $\omega_{\alpha 1}$ and $\omega_{\alpha 2}$ represent the specific humidity of the air at the inlet and outlet, respectively. The air absorbs moisture during the cooling process, leading to an increase in its humidity content and thus to water loss through evaporation.

The required air mass flow rate $\dot{m}_{\alpha}$ is linked to the thermal power that must be dissipated and the change in specific enthalpy of air between inlet and outlet [1, 2]

$$\dot{Q}_{loss} = \dot{m}_\alpha \cdot (h_{\alpha 2} - h_{\alpha 1}) \tag{4}$$

where $h_{\alpha 1}$ and $h_{\alpha 2}$ are the specific enthalpies of air at the inlet and outlet of the cooling tower respectively.

The calculation of specific humidity $\omega_{\alpha i}$ of the air is performed according to the formulation provided by Wallace [3]:

$$\omega_{\alpha i} = 0.622 \cdot \left(\frac{p_v}{101325 - p_v} \right) \tag{5}$$

with p_v being the water vapor pressure of water vapor in the air, and 101325 Pa is the standard atmospheric pressure. The vapor pressure p_v is computed as a function of the ambient temperature and relative humidity RH, using the following empirical expression [3]:

$$p_v = RH \cdot exp \left(23.709 - \frac{4111}{T_{amb} - 35.44} \right) \tag{6}$$

Where RH is obtained using the ambient and dew point temperature via [4]:

$$e_{T_D} = 6.112 \, \exp\left(\frac{17.67 \, T_D}{T_D + 243.5} \right),$$

$$e_T = 6.112 \, \exp\left(\frac{17.67 \, T}{T + 243.5} \right),$$

$$RH = 100 \times \frac{e_{T_D}}{e_T},$$

The specific enthalpy $h_{\alpha i}$ of moist air is then determined using standard psychometric relations [5]:

$$h = Cp_{air} \cdot T_{amb} + \omega_{\alpha i} \cdot (Cp_{vap} \cdot T_{amb} + h_{fg}) \tag{7}$$

where Cp_{air} is the specific heat capacity of dry air, Cp_{vap} is the specific heat capacity of water vapor, h_{fg} is the latent heat of vaporization, and T_{amb} is the ambient air temperature. Due to the temperature difference between the inlet and outlet of the cooling tower, the specific humidity and enthalpy values vary accordingly. At the outlet, the air is assumed to be saturated (i.e., 100% relative humidity), and the conditions are defined by the wet-bulb temperature plus the approach temperature [1]:

$$\omega_{\alpha 2}(T_{wb} + T_{Approach}, RH = 100\%) \tag{8}$$

$$h_{\alpha 2}(T_{wb} + T_{Approach}, \omega_{\alpha 2}) \tag{9}$$

At the inlet, the air conditions are determined by the local ambient temperature and relative humidity [1]:

$$\omega_{\alpha 1}(T_{amb}, RH_{amb}) \tag{10}$$

$$h_{\alpha 1}(T_{amb}, \omega_{\alpha 1}) \tag{11}$$

Here, T_{wb} is the local wet bulb temperature calculated using empirical relations as a function of the relative humidity and dry-bulb temperature [6]. The approach temperature $T_{Approach}$ represents the difference between the water temperature leaving the cooling tower and the ambient wet-bulb temperature. It serves as an indicator of the cooling tower's efficiency, with typical values ranging between 4–7°C [7]. In addition to evaporative losses, two other mechanisms contribute to water consumption in cooling towers. The drift losses $\dot{m}_{drift}$, which are caused by small water droplets carried away with the exhaust air stream. These are typically approximated as a fraction d of the evaporative losses [1]:

$$\dot{m}_{drift} = d \cdot \dot{m}_{evap} \tag{12}$$

The value of d depends on system design and operational conditions, and typically ranges from 0.0005% to 0.2% [5]. The second are the blow down losses $\dot{m}_{bd}$ which represent the periodic discharge of a portion of the circulating water to control the buildup of dissolved minerals and other impurities. These losses are calculated as [1]:

$$\dot{m}_{bd} = \frac{\dot{m}_{evap}}{n - 1} \tag{13}$$

where n represents the typical number of cycles and is set to $n = 3$ [5]. In summary, the amount of water consumed for cooling electrolysis using evaporative cooling towers can be phrased as a function of the local ambient dry bulb temperature T_{amb}, local relative humidity RH, and electrolyzer efficiency.

Supplementary Table 1: Extended list of parameters and constants used in the evaporative-cooling model.

**Parameter**	**Symbol**	**Value / Range**	**Unit**	**Source / Note**
Electrolyser and Heat Balance				
Hydrogen production rate	$\dot{m}_{\mathrm{H}_2}$	model variable	$\mathrm{kg\,s^{-1}}$	normalized to 1
Stack heat loss	$\dot{Q}_{\mathrm{loss}}$	see Eq.1	W	function of η_{el}, HHV
Stack efficiency	η_{el}	0.6-0.8	W	[8, 9]
Cooling Tower Parameters				
Evaporative loss	$\dot{m}_{\mathrm{evap}}$	$\propto \dot{Q}_{\mathrm{loss}}$	$\mathrm{kg\,s^{-1}}$	Eq.2
Drift loss fraction	d	0.0005–0.002	–	[5]
Cycles of concentration	n	3	–	[5]
Blow-down rate	$\dot{m}_{\mathrm{bd}}$	$\dot{m}_{\mathrm{evap}}/(n-1)$	$\mathrm{kg\,s^{-1}}$	derived
Approach temperature	ΔT_{app}	5	°C	[10]
Range temperature	$\Delta T_{\mathrm{range}}$	8–12	°C	assumed industrial default
Latent heat of vaporization	h_{fg}	2.45×10^6	$\mathrm{J\,kg^{-1}}$	at 25°C [2]
Specific heat of air	Cp_{air}	1005	$\mathrm{J\,kg^{-1}\,K^{-1}}$	[5]
Specific heat of water	Cp_{vap}	4180	$\mathrm{J\,kg^{-1}\,K^{-1}}$	[5]
Psychrometric Relations				
Saturation vapor pressure	$p_{\mathrm{sat}}(T)$	Clausius–Clapeyron	Pa	used to compute RH
Wet-bulb temperature	T_{wb}	empirical (Stull, 2011)	°C	[6]
Relative humidity	RH	ERA5 mean	%	derived from dew-point temperature
Dew-point temperature	T_{dew}	ERA5 mean	°C	[11]
Data and Forcing Inputs				
ERA5 coverage	–	2010–2020	–	10-yr climatology, 0.25° grid
Water-stress dataset	–	Aqueduct 4.0	–	[12]
Global capacity factors	–	PV, wind onshore	–	[13, 14]

Supplementary Method 2: Composite Water Risk Index

The estimation of water sustainable hydrogen production sites by water electrolysis follows values of a constructed Water Risk Index $Z_{i,s}$ shown in equation 14.

$$Z_{i,s} = \alpha_s x_{i,s} + \beta_s y_{i,s} \tag{14}$$

Here, $x_{i,s}$ denotes the water-consumption value obtained through the model in Supplementary Method 1 and $y_{i,s}$ denotes the water stress value [12] for geo-coordinate i in season s respectively. The values of α_s and β_s represent the specific weights for each parameter. For comparability, $x_{i,s}$ and $y_{i,s}$ are expressed in terms of standard deviations by computing the z-scores for each point in i and s (see eq. 15) with $\mu_{x,s}$ being the seasonal average of the considered variable Var and $\sigma_{x,s}$ being the standard deviation.

$$x_{i,s} = \frac{Var_{i,s} - \mu_{x,s}}{\sigma_{x,s}} \tag{15}$$

The determination of the weights (α_s, β_s) is performed by principal component analysis (PCA) [15, 16]. For that, an Eigen-decomposition of the weighted covariance matrix is performed (eq. 16)

$$\begin{bmatrix} s_{xx} & s_{sxy} \\ s_{syx} & s_{yy} \end{bmatrix} v_{k,s} = \lambda_{k,s} v_{k,s} \quad \text{with} \qquad \lambda_{1,s} > \lambda_{2,s} \tag{16}$$

The detailed formulation of the covariance matrix used in equation 16 is provided here. For each season s, the standardized variables $x_{i,s}$ and $y_{i,s}$ (cooling-water consumption and water stress, respectively) were arranged in an $n \times 2$ matrix $X_s = [x_{i,s}, y_{i,s}]$, where n is the number of land grid cells. The seasonal covariance matrix was then computed as

$$\Sigma_s = \frac{1}{n-1} X_s^{\mathsf{T}} X_s = \begin{bmatrix} s_{xx,s} & s_{xy,s} \\ s_{yx,s} & s_{yy,s} \end{bmatrix}, \tag{17}$$

where $s_{xx,s} = \mathrm{Var}(x_s)$, $s_{yy,s} = \mathrm{Var}(y_s)$, and $s_{xy,s} = s_{yx,s} = \mathrm{Cov}(x_s, y_s)$. Eigen-decomposition of Σ_s yields eigenvalues $\lambda_{1,s} > \lambda_{2,s}$ and corresponding normalized eigenvectors $v_{1,s} = [\alpha_s, \beta_s]^{\mathsf{T}}$. The first component (PC1) explains on average more than 80 % of the total variance across all seasons and therefore captures the dominant co-variability between modeled water consumption and water stress. The resulting weights (α_s, β_s) are positive and

typically range between 0.45-0.70. The seasonal combined z-scores $Z_{i,s}$ are aggregated into a weighted single risk index following

$$I_i = \sum_s \omega_s Z_{i,s} \quad \text{with} \quad \sum \omega_s = 1 \tag{18}$$

Weights ω_s are selected to be equal for all seasons i.e 0.25 due to nearly symmetric distribution of consumption values along time axis. For better interpretation the values are rescaled to assume numbers $\bar{I} \in [0, 100]$. The resulting scores are classified for each site into Go ($\bar{I} \in [0, 30]$), Caution ($\bar{I} \in [30, 60]$) and Other solutions ($\bar{I} \in [60, 100]$). These interval boundaries correspond approximately to the terciles of the global index distribution, ensuring that each class represents a statistically balanced share of the land surface while maintaining clear interpretability. In practical terms, values below 30 indicate locations where modeled evaporative cooling exerts little additional pressure on local water resources, scores between 30 and 60 denote regions where cooling demand and water scarcity contribute comparably to overall risk, and scores above 60 identify environments where both indicators simultaneously reach high values and where evaporative cooling would likely aggravate water stress. A larger interval for the Other solutions regions has been chosen due to the skewed distribution of the water stress indices above the median values.

Supplementary Method 3: Data processing

ERA5 reanalysis data from the Copernicus Climate Data Store were used to obtain long-term climatological means of ambient and dew-point temperature, as well as relative humidity, at a spatial resolution of 0.25° for the period 2010–2020. Monthly fields of 2 m air temperature (t2m) and dew-point temperature (d2m) were converted to seasonal and annual averages using custom-built Julia code. All climatic variables were bilinearly regridded to a global $0.25^\circ \times 0.25^\circ$ mesh to ensure consistency across datasets. The World Resources Institute (WRI) Aqueduct 4.0 dataset was resampled from its native grid to the ERA5 grid by nearest-neighbor interpolation using a custom build nearest neighbor algorithm. Water-stress classes were retained as continuous values for statistical analysis. To assess the geographical exposure of current and planned electrolysis projects, spatial coordinates from the IEA Hydrogen Database (2024) were matched to the processed grids. Project capacities were aggregated by water-stress class and seasonal cooling-water requirement to generate the global overlaps shown in the main text. All spatial analysis were performed using custom-built Julia code.

Supplementary Notes

Supplementary Note 1: Model sensitivities

To quantify parameter uncertainty in the evaporative cooling model, we performed a two-part sensitivity analysis. First, we evaluate the response of specific cooling water consumption to variations in electrolyzer efficiency across representative climate regimes (Supplementary Table 2, Supplementary Figure 1). Second, we rank the relative importance of the remaining model parameters using a one-at-a-time (OAT) sensitivity analysis (Supplementary Figure 2).

Supplementary Table 2: Cooling water demand across climate archetypes for different electrolyser efficiencies. Values are given in L/kg H_2 and include cooling only, excluding feedstock water.

**Climate archetype**	$\eta = 0.60$	$\eta = 0.65$	$\eta = 0.70$	$\eta = 0.75$	$\eta = 0.80$
Hot/dry (desert)	33.69	26.94	21.15	16.14	11.75
Hot/humid (tropical)	29.13	23.27	18.25	13.90	10.10
Temperate	25.69	20.49	16.03	12.17	8.79
Cool/humid (Nordic)	23.04	18.36	14.35	10.87	7.83
Cold/arid (steppe)	22.15	17.64	13.78	10.43	7.50

Five representative climate archetypes were selected to span the global ERA5 distribution of ambient and dew-point conditions: hot-dry (35 °C / 12 % RH), hot-humid (30 °C / 75 % RH), temperate (20 °C / 60 % RH), cool-humid (8 °C / 80 % RH), and cold-arid (5 °C / 35 % RH). For each archetype, specific cooling water consumption was evaluated at five efficiency values, $\eta_{\text{el}} \in \{0.60, 0.65, 0.70, 0.75, 0.80\}$, covering the realistic industrial range considered in this study.

Supplementary Figure 1a shows that the absolute spread between climate archetypes is substantial, ranging from approximately $22\,L\,\text{kg}_{\text{H}_2}^{-1}$ in cold-arid conditions to $34\,L\,\text{kg}_{\text{H}_2}^{-1}$ in hot-dry conditions at $\eta_{\text{el}} = 0.60$. At higher efficiencies, these values converge to approximately 7–12 $L\,\text{kg}_{\text{H}_2}^{-1}$ at $\eta_{\text{el}} = 0.80$. In contrast, the relative response shown in Supplementary Figure 1b collapses nearly all archetypes onto a single curve, indicating that changes in efficiency act as an almost climate-invariant multiplicative scaling of cooling demand. Across all archetypes, specific consumption increases by roughly

25 % at $\eta_{\text{el}} = 0.60$ and decreases by roughly 45 % at $\eta_{\text{el}} = 0.80$ relative to the baseline case of $\eta_{\text{el}} = 0.65$. Panels (c) and (d) confirm that this pattern is preserved when idealized archetypes are replaced by the full distribution of ERA5 land grid cells.

This behavior has a direct implication for the spatial risk classification presented in the main manuscript. The classification of grid cells into Go, Caution, and Other Solutions categories is based on the z-standardized cooling water consumption field $x_{i,s}$. Let $\tilde{x}_{i,s} = c\,x_{i,s}$ denote the consumption field after uniform multiplicative rescaling by a factor $c > 0$, as induced for example by a change in electrolyzer efficiency. Because z-scores are invariant under positive affine rescaling,

$$\tilde{z}_{i,s} = \frac{\tilde{x}_{i,s} - \mu_{\tilde{x},s}}{\sigma_{\tilde{x},s}} = \frac{c\,x_{i,s} - c\,\mu_{x,s}}{c\,\sigma_{x,s}} = z_{i,s}, \tag{19}$$

the standardized cooling-water field remains unchanged. As a consequence, the PCA-derived weights (α_s, β_s) and the resulting risk index I_i are unaffected by parameter variations that rescale all grid cells proportionally. This applies not only to electrolyzer efficiency, but also to parameters such as higher heating value, drift losses, and blow-down losses. These parameters therefore modify absolute cooling water consumption values without altering the relative spatial pattern of risk shown in the main manuscript. Recomputing the global maps at alternative parameter values would thus reproduce the same classification pattern by construction.

To complement the efficiency analysis, we further performed an OAT sensitivity analysis across the remaining model parameters over their respective realistic ranges (Supplementary Table 1), evaluated for two contrasting ERA5 climate cells: a temperate case (20 °C / 60 % RH) and a hot-dry case (35 °C / 12 % RH). The results are summarized in the tornado chart in Supplementary Figure 2.

Electrolyzer efficiency η_{el} remains the dominant driver of model output in both climates, with total swings of approximately $\Delta \approx 18\,L\,\text{kg}_{\text{H}_2}^{-1}$ in the temperate case and $\Delta \approx 22\,L\,\text{kg}_{\text{H}_2}^{-1}$ in the hot-dry case. Cycles of concentration n rank second, with $\Delta \approx 6\text{-}7\,L\,\text{kg}_{\text{H}_2}^{-1}$, followed by the drift loss fraction d. Parameters related to cooling-tower thermal design, such as the cooling range, exert comparatively smaller and more climate-dependent effects.

Overall, the OAT ranking supports two conclusions relevant to the manuscript. First, the electrolyzer efficiency parameter is the principal source of model uncertainty, which justifies the dedicated analysis above. Second, the con-

tribution of secondary parameters remains limited compared with the much larger inter-regional spread imposed by climatological forcing itself, which reaches on the order of $20\,L\,\mathrm{kg}_{\mathrm{H_2}}^{-1}$ between cool/humid and hot/dry environments. The central spatial conclusions of the study are therefore robust to plausible variation in model parameterization.

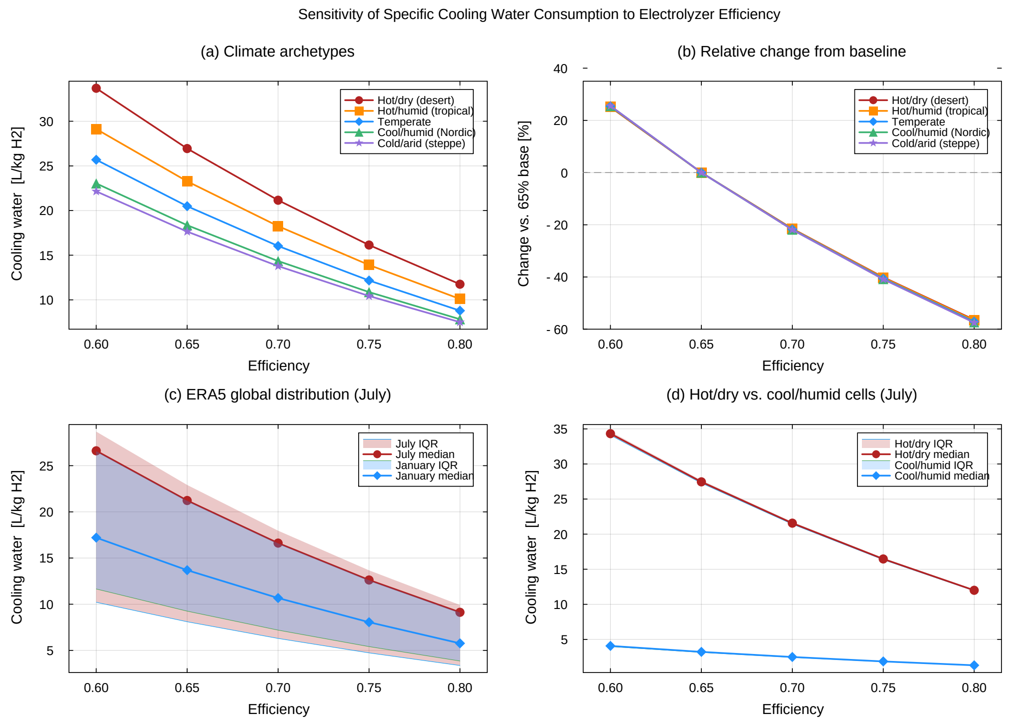

Supplementary Figure 1: Sensitivity of specific cooling-water consumption to electrolyser efficiency. **a,** Specific cooling-water consumption ($L_{H_2O}\,kg_{H_2}^{-1}$) as a function of electrolyser efficiency ($\eta_{el} \in [0.60, 0.80]$) for five representative climate archetypes spanning the global ERA5 distribution: hot-dry (desert, 35 °C / 12% relative humidity), hot-humid (tropical, 30 °C / 75% relative humidity), temperate (20 °C / 60% relative humidity), cool-humid (Nordic, 8 °C / 80% relative humidity), and cold-arid (steppe, 5 °C / 35% relative humidity). **b,** Relative change in specific cooling-water consumption with respect to the baseline case $\eta_{el} = 0.65$. The near-perfect collapse of all archetypes onto a single curve demonstrates that efficiency acts as an almost climate-invariant multiplicative scaling of consumption, ranging from approximately +25% at $\eta_{el} = 0.60$ to approximately −45% at $\eta_{el} = 0.80$. **c,** Empirical distribution of specific cooling-water consumption across all ERA5 land grid cells for July and January. Lines show median values and shaded bands show interquartile ranges. **d,** Comparison of hot-dry and cool-humid ERA5 grid cells for July. Lines show median values and shaded bands show interquartile ranges, illustrating that the absolute spread between climates increases toward lower efficiencies while the relative sensitivity remains nearly unchanged.

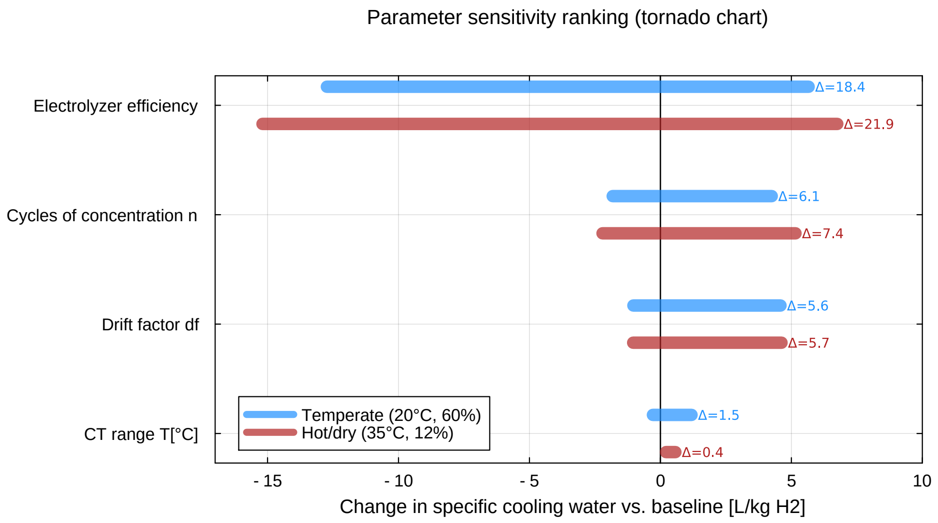

Supplementary Figure 2: One-at-a-time sensitivity of specific cooling-water consumption to model parameters. Specific cooling-water consumption is given in $L_{\mathrm{H_2O}}\,\mathrm{kg}_{\mathrm{H_2}}^{-1}$ and evaluated for two representative ERA5 climate cells: temperate (20 °C / 60% relative humidity, blue bars) and hot-dry (35 °C / 12% relative humidity, red bars). Each bar shows the absolute change in consumption obtained when one parameter is swept across its realistic range (Supplementary Table 1) while all others are held at baseline values. Δ labels denote the total swing, defined as the maximum minus minimum consumption across the tested range. Electrolyser efficiency (η_{el}) dominates the sensitivity in both climates, followed by cycles of concentration (n). Parameters associated with cooling-tower design exhibit smaller and more climate-dependent effects, indicating that their influence on global results is secondary to the efficiency-driven heat rejection.

Supplementary Note 2: Energy and cost penalty of seawater desalination for cooling-water supply

This section quantifies the incremental energy and cost penalty associated with supplying the cooling water demand derived in Section 2.1 of the main manuscript via seawater reverse osmosis (SWRO). The calculation is expressed as an incremental contribution on top of the approximately 10 L kg^{-1} H_2 of reaction water that is already implicitly included in standard hydrogen cost estimates [17].

Input parameters

The following ranges are adopted for the calculation (Supplementary Table 3). Cooling water demand is taken directly from the thermodynamic model results of Section 2.1. SWRO specific energy consumption and levelized cost of water correspond to the commercial range consistently reported across independent techno-economic reviews. The brine-management surcharge was discussed in Section 3 of the main manuscript and is included here for completeness. System-level electrolysis energy consumption and current levelized cost of hydrogen are used as reference quantities for expressing the penalty as a fractional increase.

Incremental energy penalty

The incremental energy demand for desalinating the cooling-water supply is given by

$$E_{\text{desal}} = V_{\text{cool}} \cdot \text{SEC}_{\text{SWRO}} \cdot 10^{-3}, \tag{20}$$

where V_{cool} is the cooling-water demand in L kg^{-1} H_2, SEC_{SWRO} is the SWRO specific energy consumption in kWh m^{-3}, and the factor 10^{-3} converts from liters to cubic meters. Applied to the ranges in Supplementary Table 3:

$$E_{\text{desal,low}} = 20 \cdot 3 \cdot 10^{-3} = 0.060\ \text{kWh}\,\text{kg}^{-1}\,\text{H}_2, \tag{21}$$

$$E_{\text{desal,high}} = 40 \cdot 5 \cdot 10^{-3} = 0.200\ \text{kWh}\,\text{kg}^{-1}\,\text{H}_2. \tag{22}$$

Rounded to account for upper-range SEC uncertainty, the incremental energy penalty is reported as $E_{\text{desal}} \approx$ 0.08–0.25 kWh kg^{-1} H_2.

Supplementary Table 3: Input parameters for the desalination penalty calculation.

**Quantity**	**Low**	**High**	**Source**
Incremental cooling water demand [L kg^{-1} H_2]	20	40	This work, Section 2.1
Reference reaction water demand [L kg^{-1} H_2]	9	10	[17]
SWRO specific energy consumption [kWh m^{-3}]	3	5	[18, 17, 19]
SWRO levelized cost of water [USD m^{-3}]	0.7	1.5	[18, 19, 17]
Brine-management surcharge [USD m^{-3}]	0.3	0.7	[20]
System-level electrolysis energy [kWh kg^{-1} H_2]	50	55	[21]
Cost optimized LCOH [USD kg^{-1} H_2]	2	6	[22]

Expressed as a fractional increase relative to the system-level electrolysis energy consumption E_{el} = 50–55 kWh kg^{-1} H_2 [21]:

$$\frac{E_{\mathrm{desal}}}{E_{\mathrm{el}}} \approx 0.15\text{–}0.50\ \%. \tag{23}$$

Incremental cost penalty

The incremental cost penalty combines the SWRO levelized cost of water with the brine-management surcharge:

$$C_{\mathrm{desal}} = V_{\mathrm{cool}} \cdot (\mathrm{LCOW}_{\mathrm{SWRO}} + C_{\mathrm{brine}}) \cdot 10^{-3}. \tag{24}$$

Applied to the ranges in Supplementary Table 3:

$$C_{\mathrm{desal,low}} = 20 \cdot (0.7 + 0.3) \cdot 10^{-3} = 0.020\ \mathrm{USD\,kg^{-1}\,H_2}, \tag{25}$$

$$C_{\mathrm{desal,high}} = 40 \cdot (1.5 + 0.7) \cdot 10^{-3} = 0.088\ \mathrm{USD\,kg^{-1}\,H_2}. \tag{26}$$

The incremental cost penalty is therefore $C_{\mathrm{desal}} \approx$ 0.02–0.09 USD kg^{-1} H_2. Expressed as an incremental fractional increase relative to the cost-optimized

levelized cost of hydrogen from hybrid solar PV and onshore wind production reported by the IEA Global Hydrogen Review 2024 for the Net Zero Emissions by 2050 Scenario in 2030 (2–6 USD kg^{-1} H_2, [22], Figure 3.13):

$$\frac{C_{\mathrm{desal}}}{\mathrm{LCOH}_{\mathrm{IEA\,2030}}} \approx 0.3\text{–}4.5\ \%. \tag{27}$$

Benchmark against reaction-water-only assessments

Previous assessments of the desalination penalty for green hydrogen have considered only the stoichiometric reaction-water demand of approximately 10 L kg^{-1} H_2. Beswick et al. [17] report an incremental energy overhead of less than 0.13 % of the minimum electrolysis energy demand and a cost overhead of less than 0.01 USD kg^{-1} H_2, concluding that water supply is not a binding constraint for electrolyzer deployment. An independent analysis by Khan et al. [23] reaches equivalent results, reporting an energy overhead of approximately 0.1 % and a cost overhead of approximately 0.02 USD kg^{-1} H_2.

Applying Eqs. 20 and 24 to the reaction-water volume of 10 L kg^{-1} H_2 reproduces these values: $E^{\mathrm{rxn}}_{\mathrm{desal}} \approx$ 0.03 -0.05 kWh kg^{-1} H_2 (0.06–0.10 % of electrolysis energy) and $C^{\mathrm{rxn}}_{\mathrm{desal}} \approx$ 0.01–0.02 USD kg^{-1} H_2, in agreement with the published literature. Including the additional cooling-water demand of 20–40 L kg^{-1} H_2 quantified in this study therefore amplifies both the energy and cost penalties of the desalination pathway by approximately a factor of three to five.

Supplementary References

[1] Ifeanyi Njoku and Ogheneruona Diemuodeke. Techno-economic comparison of wet and dry cooling systems for combined cycle power plants in different climatic zones. *Energy Conversion and Management*, 227:113610, jan 2021.

[2] W. P. Jones. *Air Conditioning Engineering*. Routledge, 5th edition, 2001.

[3] John M. Wallace and Peter V. Hobbs. *Atmospheric Science: An Introductory Survey*. Academic Press, San Diego, 2nd edition, 2006.

[4] D. Bolton. The computation of equivalent potential temperature. *Monthly Weather Review*, 108(7):1046–1053, 1980.

[5] American Society of Heating, Refrigerating and Air-Conditioning Engineers. *2017 ASHRAE Handbook—Fundamentals*. American Society of Heating, Refrigerating and Air-Conditioning Engineers, Atlanta, GA, 2017. Chapter on psychrometrics.

[6] Roland Stull. Wet-bulb temperature from relative humidity and air temperature. *Journal of Applied Meteorology and Climatology*, 50:2267–2269, 11 2011.

[7] Don Eppelheimer and Brenda Bradley. Selecting Cooling Towers for Efficiency—Range or Approach? *Trane Engineers Newsletter*, 34(1), January 2005. Accessed: 2025-04-04.

[8] Peter Ellersdorfer, Amr Omar, Isabelle Rider, Rahman Daiyan, and Greg Leslie. The hydrogen-water collision: Assessing water and cooling demands for large-scale green hydrogen production in a warming climate. *International Journal of Hydrogen Energy*, 97:1002–1013, 2025.

[9] Peter Ellersdorfer, Amr Omar, Robert A. Taylor, Rahman Daiyan, and Greg Leslie. Multi-effect distillation: a sustainable option to large-scale green hydrogen production using solar energy. *International Journal of Hydrogen Energy*, 48(81):31491–31505, 2023.

[10] Mick Schwedler. Effect of heat rejection load and wet bulb on cooling tower performance. *ASHRAE Journal*, 56(1):18–28, jan 2014.

[11] Copernicus Climate Change Service. ERA5 Reanalysis: Single Levels. https://cds.climate.copernicus.eu/datasets/reanalysis-era5-single-levels?tab=overview, 2023. Accessed: March 9, 2025.

[12] World Resources Institute. Aqueduct 4.0: Updated Decision-Relevant Global Water Risk Indicators. https://www.wri.org/research/aqueduct-40-updated-decision-relevant-global-water-risk-indicators, 2023. Accessed: March 9, 2025.

[13] S. Ishmam, H. Heinrichs, C. Winkler, B. Bayat, A. Lahnaoui, S. Agbo, E.U. Pena Sanchez, D. Franzmann, N. Oijeabou, C. Koerner, Y. Michael, B. Oloruntoba, C. Montzka, H. Vereecken, H. Hendricks Franssen, J. Brendt, S. Brauner, W. Kuckshinrichs, S. Venghaus, D. Kone, B. Korgo, K. Ogunjobi, V. Chiteculo, J. Olwoch, Z. Getenga, J. Linßen, and D. Stolten. Mapping local green hydrogen cost-potentials by a multidisciplinary approach. *International Journal of Hydrogen Energy*, 87:1155–1170, 2024.

[14] Edgar Ubaldo Peña-Sánchez, Philipp Dunkel, Christoph Winkler, Heidi Heinrichs, Florian Prinz, Jann Weinand, Rachel Maier, Sebastian Dickler, Shuying Chen, Katharina Gruber, Theresa Klütz, Jochen Linßen, and Detlef Stolten. Towards High Resolution, Validated and Open Global Wind Power Assessments. *arXiv preprint*, 2025.

[15] Ian T. Jolliffe. *Principal Component Analysis*. Springer Series in Statistics. Springer, 2nd edition, 2002.

[16] Hervé Abdi and Lynne J. Williams. Principal component analysis. *Wiley Interdisciplinary Reviews: Computational Statistics*, 2(4):433–459, 2010.

[17] Rebecca R. Beswick, Alexandra M. Oliveira, and Yushan Yan. Does the green hydrogen economy have a water problem? *ACS Energy Letters*, 6(9):3167–3169, 2021.

[18] Upeksha Caldera and Christian Breyer. Learning curve for seawater reverse osmosis desalination plants: Capital cost trend of the past, present, and future. *Water Resources Research*, 53(12):10523–10538, 2017.

[19] Pranjal Kumar, Abhijit Date, Nasir Mahmood, Ratan Kumar Das, and Bahman Shabani. Freshwater supply for hydrogen production: An

underestimated challenge. *International Journal of Hydrogen Energy*, 78:202–217, 2024.

[20] D. Ghernaout. Environmental Impacts of Desalination and Brine Treatment: Challenges and Mitigation Measures. *Open Access Library Journal*, 2020.

[21] Alexander Buttler and Hartmut Spliethoff. Current status of water electrolysis for energy storage, grid balancing and sector coupling via power-to-gas and power-to-liquids: A review. *Renewable and Sustainable Energy Reviews*, 82:2440–2454, 2018.

[22] International Energy Agency. Global Hydrogen Review 2024. Technical report, International Energy Agency, Paris, 2024. Accessed: 2025-04-04.

[23] M. A. Khan, Tareq Al-Attas, Soumyabrata Roy, Muhammad M. Rahman, Noreddine Ghaffour, Venkataraman Thangadurai, Stephen Larter, Jinguang Hu, Pulickel M. Ajayan, and Md Golam Kibria. Seawater electrolysis for hydrogen production: a solution looking for a problem? *Energy & Environmental Science*, 14:4831–4839, 2021.
