# Supplementary material for: Demand for cooling water reshapes global water-sustainable hydrogen production
Source: Commun Sustain. 2026 Jun 26;1(1):103. doi: 10.1038/s44458-026-00106-x (PMC13303071; doi:10.1038/s44458-026-00106-x)
Supplement: Supplementary file 1 — Transparent Peer Review file [file 44458_2026_106_MOESM1_ESM.pdf]

nature portfolio

Peer Review File

Demand for cooling water reshapes global water-sustainable hydrogen production

Corresponding Author: Mr Bernhard Wortmann

This file contains all editorial decision letters in order by version, followed by all author rebuttals in order by version.

Attachments originally included by the reviewers as part of their assessment can be found at the end of this file.

Version 0:

Decision Letter:

** Please ensure you delete the link to your author home page in this e-mail if you wish to forward it to your coauthors **

Dear Mr Wortmann,

Your manuscript titled "Cooling Water Demand Reshapes Global Water-Sustainable Hydrogen Production" has now been seen by 2 reviewers, and we include their comments at the end of this message. They find your work of interest, but some important points are raised. We are interested in the possibility of publishing your study in Communications Sustainability, but would like to consider your responses to these concerns and assess a revised manuscript before we make a final decision on publication.

In particular, please ensure that the revised manuscript meets the following editorial thresholds:
E1. Provide additional robust evidence and validated insights into global water consumption for PEM and alkaline electrolysis, while accounting for alternative water sources and technologies (e.g., desalination).
E2. Fully describe and justify all critical parameters, including electrolyser efficiency and evaporation, and include an appropriate parameter study (e.g., sensitivity analysis).

We therefore invite you to revise and resubmit your manuscript, along with a point-by-point response that takes into account the points raised. Please highlight all changes in the manuscript text file.

Please submit your point-by-point responses as a separate file, distinct from your cover letter where you can add responses to the Editors' comments that you do not want to be made available to the reviewers. Word files are preferred. We recommend that any figures, tables or graphs that are included in the response to reviewers are also included in the main article or Supplementary Information.

We are committed to providing a fair and constructive peer-review process. Please don't hesitate to contact us if you wish to discuss the revision in more detail.

Please use the following link to submit your revised manuscript, point-by-point response to the reviewers' comments (which should be in a separate document to any cover letter), a tracked-changes version of the manuscript (as a PDF file) and the completed checklist:
Link Redacted
** This url links to your confidential home page and associated information about manuscripts you may have submitted or be reviewing for us. If you wish to forward this email to co-authors, please delete the link to your homepage first **

We hope to receive your revised paper within six weeks; please let us know if you aren't able to submit it within this time so that we can discuss how best to proceed. If we don't hear from you, and the revision process takes significantly longer, we may close your file. In this event, we will still be happy to reconsider your paper at a later date, as long as nothing similar has been accepted for publication at Communications Sustainability or published elsewhere in the meantime.

Please do not hesitate to contact us if you have any questions or would like to discuss these revisions further. We look forward to seeing the revised manuscript and thank you for the opportunity to review your work.

Best regards,

Lukas Gast, PhD
Editorial Board Member
Communications Sustainability

Nicola Colombo, PhD
Associate Editor, Communications Earth & Environment
Consulting Editor, Communications Sustainability

EDITORIAL POLICIES AND FORMATTING

We ask that you ensure your manuscript complies with our editorial policies. Please ensure that the following formatting requirements are met, and any checklist relevant to your research is completed and uploaded as a Related Manuscript file type with the revised article.

For Manuscripts that fall into the following fields:
• Behavioural and social science
• Ecological, evolutionary & environmental sciences
• Life sciences
An updated and completed version of our Reporting Summary must be uploaded with the revised manuscript
You can download the form here:
https://www.nature.com/documents/nr-reporting-summary.pdf

Furthermore, please align your manuscript with our format requirements, which are summarized on the following checklist:
Communications Sustainability formatting checklist

and also in our style and formatting guide Communications Sustainability formatting guide .

*** DATA: Communications Sustainability endorses the principles of the Enabling FAIR data project (http://www.copdess.org/enabling-fair-data-project/). We ask authors to make the data that support their conclusions available in permanent, publicly accessible data repositories. (Please contact the editor if you are unable to make your data available).

All Communications Sustainability manuscripts must include a section titled "Data Availability" at the end of the Methods section or main text (if no Methods). More information on this policy, is available at http://www.nature.com/authors/policies/data/data-availability-statements-data-citations.pdf.

In particular, the Data availability statement should include:
- Unique identifiers (such as DOIs and hyperlinks for datasets in public repositories)
- Accession codes where appropriate
- If applicable, a statement regarding data available with restrictions
- If a dataset has a Digital Object Identifier (DOI) as its unique identifier, we strongly encourage including this in the Reference list and citing the dataset in the Data Availability Statement.

DATA SOURCES: All new data associated with the paper should be placed in a persistent repository where they can be freely and enduringly accessed. We recommend submitting the data to discipline-specific, community-recognized repositories, where possible and a list of recommended repositories is provided at http://www.nature.com/sdata/policies/repositories.

If a community resource is unavailable, data can be submitted to generalist repositories such as figshare or Dryad Digital Repository. Please provide a unique identifier for the data (for example a DOI or a permanent URL) in the data availability statement, if possible. If the repository does not provide identifiers, we encourage authors to supply the search terms that will return the data. For data that have been obtained from publicly available sources, please provide a URL and the specific data product name in the data availability statement. Data with a DOI should be further cited in the methods reference section.

Please refer to our data policies at http://www.nature.com/authors/policies/availability.html.

REVIEWER COMMENTS:

Reviewer #1 (Remarks to the Author):

This manuscript addresses a critical and understudied topic in green hydrogen development: the global spatial heterogeneity of cooling water demand and its coupling with water scarcity risks for electrolytic hydrogen production. The integrated analytical framework combining a thermodynamic evaporative cooling model with a composite water risk index is novel, and the findings provide valuable insights for the spatial planning and policy design of the global hydrogen economy, aligning well with the journal's focus on sustainability research. However, the manuscript has notable gaps in model validation, parameter analysis, technical differentiation, and empirical support that require substantial revisions and supplementary analyses.

1. The thermodynamic evaporative cooling model relies on fixed values/empirical ranges for critical parameters (e.g., electrolyser efficiency = 0.65, cooling tower approach temperature = 3–5 °C, cycles of concentration n = 3; Table 1 in main text, Table S1 in SI). The manuscript fails to quantify how fluctuations in these parameters impact the calculated cooling water consumption (LH2O /kg H2), nor does it conduct a systematic sensitivity analysis. For instance, electrolyser efficiency varies between 0.6–0.8 in industrial applications, which would directly alter stack heat loss (Qloss) and subsequent evaporative water demand.
2. The manuscript focuses on both PEM and alkaline electrolysers but does not distinguish their inherent differences in cooling water characteristics in the thermodynamic model and subsequent analyses. In practice, PEM electrolysers operate at lower temperatures (80–90°C) compared to alkaline electrolysers (100–150°C), leading to distinct heat dissipation profiles and cooling water requirements.
3. The manuscript notes that green hydrogen projects in arid regions must adopt non-freshwater sources (e.g., seawater desalination) but only briefly mentions that desalination is "technically feasible but faces brine management challenges." It fails to quantify the energy, cost, and water footprint impacts of non-freshwater sources for evaporative cooling, nor does it integrate desalination/reclaimed water into the water risk index framework. This makes the recommendation of "alternative cooling strategies" lack quantitative support.
4. The model is based on long-term climatological averages (2010–2020) from ERA5 reanalysis data and does not account for the impacts of short-term climate extremes (e.g., heatwaves, droughts) on cooling water demand. In actual operation, extreme high temperatures can significantly increase evaporative cooling requirements, exacerbating local water supply-demand imbalances. Ignoring this factor leads to a gap between model results and real operational scenarios.
5. Some figures have incomplete annotations (e.g., unlabeled vertical axis scales in Fig. 4, confusing subplot labels in Fig. 7). Standardize all figure formats, supplement missing scales, units, and legend explanations to meet the journal's publication requirements.

Reviewer #2 (Remarks to the Author):

This manuscript determines the cooling water needs in evaporative cooling towers used in hydrogen production systems using water electrolysis. It then contextualises this water need with water scarcity factor and renewable capacity factors. This topic is of significant relevance, and the mapping of risk factors is highly instructive and of interest to the wider readership in sustainability.

My main concern with this manuscript is that it focuses only on evaporative cooling towers. It seems to disregard other options that address the water scarcity concerns and balances cost of cooling systems. Although it is reasonable that once through and dry cooling systems are not analysed the option of adiabatic cooling is ignored, where cooling is for the main part a dry cooler and water is only used when ambient temperature reaches a threshold and when there is a suitable difference in dry and wet bulb temperatures. This approach can lead to smaller and lower cost cooling system than dry cooling and lower water use than evaporative cooling towers and in some regions a reasonable balance between water use and cost.

Whereas industry appears to already understand the issue of high-water demand in evaporative cooling towers and its impact in areas where water is scarce. In Australia multiple industry projects have reported that they select adiabatic cooling that has a much lower aggregate water demand. The available industry reports in Australia from ARENA knowledge sharing site (1) are of industry projects after the cited ARUP 2022 report in this manuscript (Reference 3, that appears to also be reference 15) that also ignores the adiabatic cooling option. As such I question whether this manuscript is analysing an industry problem that has already been solved.

I recommend this manuscript is revised to also include adiabatic cooling that appears to be what industry is considering for water scarce countries.

(1) https://arena.gov.au/knowledge-bank/?technology=hydrogen&item_type=Reports&state=

** Visit Nature Portfolio's author and reviewers' website at www.nature.com/authors for information about policies, services and author benefits**

Communications Sustainability is committed to improving transparency in authorship. As part of our efforts in this direction, we are now requesting that all authors identified as 'corresponding author' create and link their Open Researcher and Contributor Identifier (ORCID) with their account on the Manuscript Tracking System prior to acceptance. ORCID helps the

scientific community achieve unambiguous attribution of all scholarly contributions. You can create and link your ORCID from the home page of the Manuscript Tracking System by clicking on ‘Modify my Springer Nature account’ and following the instructions in the link below. Please also inform all co-authors that they can add their ORCIDs to their accounts and that they must do so prior to acceptance.
https://www.springernature.com/gp/researchers/orcid/orcid-for-nature-research

For more information please visit http://www.springernature.com/orcid

If you experience problems in linking your ORCID, please contact the Platform Support Helpdesk.

Version 1:

Decision Letter:

** Please ensure you delete the link to your author home page in this e-mail if you wish to forward it to your coauthors **

Dear Mr Wortmann,

Your manuscript titled "Cooling Water Demand Reshapes Global Water-Sustainable Hydrogen Production" has now been seen by our reviewers, whose comments appear below. In light of their advice we are delighted to say that we are happy, in principle, to publish a suitably revised version in Communications Sustainability.

We therefore invite you to edit your manuscript to comply with our format requirements and to maximise the accessibility and therefore the impact of your work.

EDITORIAL REQUESTS:

Please review our specific editorial comments and requests regarding your manuscript in the attached "Editorial Requests Table".

*****Please take care to match our formatting and policy requirements. We will check revised manuscript and return manuscripts that do not comply. Such requests will lead to delays. *****

Please outline your response to each request in the right hand column. Please upload the completed table with your manuscript files as a Related Manuscript file.

If you have any questions or concerns about any of our requests, please do not hesitate to contact me.

SUBMISSION INFORMATION:

In order to accept your paper, we require the files listed at the end of the Editorial Requests Table; the list of required files is also available at https://www.nature.com/documents/commsj-file-checklist.pdf .

OPEN ACCESS:

Communications Sustainability is a fully open access journal. Articles are made freely accessible on publication. For further information about article processing charges, open access funding, and advice and support from Nature Portfolio, please visit https://www.nature.com/commssustain/open-access

At acceptance, you will be provided with instructions for completing the open access licence agreement on behalf of all authors. This grants us the necessary permissions to publish your paper. Additionally, you will be asked to declare that all required third party permissions have been obtained, and to provide billing information in order to pay the article-processing charge (APC).

Please use the following link to submit the above items:
Link Redacted
** This url links to your confidential home page and associated information about manuscripts you may have submitted or be reviewing for us. If you wish to forward this email to co-authors, please delete the link to your homepage first **

We hope to hear from you within two weeks; please let us know if you need more time.

Best regards,

Lukas Gast, PhD
Editorial Board Member
Communications Sustainability

Nicola Colombo
Consulting Editor, Communications Sustainability
Associate Editor, Communications Earth & Environment

REVIEWERS' COMMENTS:

Reviewer #1 (Remarks to the Author):

The authors have addressed the reviewer's concerns well. I don't have further comments.

Reviewer #2 (Remarks to the Author):

Thank you for addressing the concerns raised in my review. The response to them is reasonable and the changes to the manuscript are acceptable. I have no further concerns about the paper. I recommend the manuscript is published.

** Visit Nature Portfolio's author and reviewers' website at www.nature.com/authors for information about policies, services and author benefits**

Open Access This Peer Review File is licensed under a Creative Commons Attribution 4.0 International License, which permits use, sharing, adaptation, distribution and reproduction in any medium or format, as long as you give appropriate credit to the original author(s) and the source, provide a link to the Creative Commons license, and indicate if changes were made.
In cases where reviewers are anonymous, credit should be given to 'Anonymous Referee' and the source.
The images or other third party material in this Peer Review File are included in the article's Creative Commons license, unless indicated otherwise in a credit line to the material. If material is not included in the article's Creative Commons license and your intended use is not permitted by statutory regulation or exceeds the permitted use, you will need to obtain permission directly from the copyright holder.
To view a copy of this license, visit https://creativecommons.org/licenses/by/4.0/

Dear Editor, dear Reviewers,

We sincerely thank you for the careful evaluation of our manuscript and for the constructive and insightful comments. We greatly appreciate the time and effort invested in reviewing our work. Your feedback has helped us to improve the manuscript substantially in terms of clarity, methodological transparency, and discussion of the broader practical implications.

In the revised version, we have carefully addressed all comments raised by the reviewers and editor. In particular, we expanded the Supplementary Information with a dedicated sensitivity analysis and a quantitative assessment of desalination-related energy and cost penalties, clarified the scope and rationale of the technology-agnostic cooling model, strengthened the discussion of adiabatic cooling and alternative water-supply strategies, and improved the presentation and annotation of the figures. A detailed point-by-point response is provided below, together with a summary of the corresponding changes made in the manuscript and Supplementary Information.

We hope that the revisions adequately address the concerns raised and that the manuscript is now suitable for publication.

Yours faithfully,
Bernhard Wortmann
PhD candidate, on behalf of all co-authors

**No.**	**Comment**	**Response**	**Changes**
	**Reviewer 1**		
1	The thermodynamic evaporative cooling model relies on fixed values/empirical ranges for critical parameters (e.g., electrolyser efficiency = 0.65, cooling tower approach temperature = 3–5 °C, cycles of concentration n = 3; Table 1 in main text, Table S1 in SI). The manuscript fails to quantify how fluctuations in these parameters impact the calculated cooling water	We thank the reviewer for this constructive suggestion. We agree that a quantitative sensitivity analysis improves the transparency and interpretability of the model. In response, we have added a dedicated new section to the Supplementary Information (Section S4, "Model Sensitivities"), which provides two complementary analyses.<br>First, we quantify the response of specific cooling water consumption to electrolyzer efficiency across the industrially relevant range of $\eta_{el} \in [0.60, 0.80]$ for five representative climate archetypes spanning the global ERA5 distribution, from	• Added dedicated SI Section 4 "*Model Sensitivities*" with two-part sensitivity analysis.<br>• Added new Table S2 reporting cooling water demand across five climate archetypes (hot-dry, hot-humid, temperate, cool-humid, cold-arid) for

	consumption (LH2O /kg H2), nor does it conduct a systematic sensitivity analysis. For instance, electrolyser efficiency varies between 0.6–0.8 in industrial applications, which would directly alter stack heat loss (Qloss) and subsequent evaporative water demand.	hot-dry desert to cold-arid steppe conditions (new Fig. S1a). The results show that cooling water consumption changes by approximately +25% at η_el = 0.60 and −45% at η_el = 0.80 relative to the baseline value of 0.65 used in the main text. As shown in Fig. S1b, this relative sensitivity is nearly climate-invariant: the curves for all five climate archetypes collapse onto an almost identical trajectory, indicating that efficiency primarily acts as a multiplicative scaling of cooling demand rather than as a climate-dependent driver. Panels S1c–d confirm that the same pattern holds when the analysis is extended from idealized archetypes to the full ERA5 land-grid distribution.<br>Second, we added a one-at-a-time tornado analysis (new Fig. S2) to rank the relative importance of the main model parameters for two contrasting ERA5 climate cells (temperate and hot-dry). This analysis confirms that electrolyzer efficiency is the dominant source of model uncertainty ($\Delta \approx 18–22$ L kg^{-1} H_2), followed by cycles of concentration and the drift loss fraction. Parameters related to cooling-tower design exhibit comparatively smaller and more climate-dependent effects, and do not alter the qualitative spatial conclusions of the manuscript.<br>We would further like to note that the spatial classification of grid cells into the Go, Caution, and Other Solutions categories is invariant under uniform multiplicative shifts in modeled cooling water consumption. As now shown explicitly in the new SI section, the Risk Index is derived from z-standardized consumption values, and z-scores are invariant under positive affine transformations (Eq. 19 in the revised Supplementary Information). Consequently, parameter variations that affect all grid cells proportionally, including electrolyzer efficiency, HHV, and drift or blow-down losses, rescale absolute cooling water consumption values without changing the relative spatial risk pattern shown in Fig. 5 of the	electrolyser efficiencies η ∈ {0.60, 0.65, 0.70, 0.75, 0.80}.<br>• Added new Figure S1 (four-panel): (a) efficiency sensitivity across climate archetypes, (b) relative change from baseline showing climate-invariant multiplicative scaling, (c) ERA5 global distribution for July and January, (d) hot-dry vs. cool-humid ERA5 cells.<br>• Added new Figure S2: tornado chart ranking four model parameters (electrolyzer efficiency, cycles of concentration, drift factor, CT range) across temperate and hot-dry climate cells.<br>• Added explicit invariance proof (Eq. 19 in SI) showing that z-standardized Risk Index is invariant under uniform multiplicative rescaling of consumption — demonstrates that parameter variations affecting all grid cells

		main text. Recomputing global maps for each tested parameter value would therefore reproduce the same spatial classification by construction.<br>For clarity, we have also added a short paragraph in Section 4.1 of the main text directing readers to the new Supplementary Information section, where the full sensitivity analysis and tested parameter ranges are now documented.	proportionally do not alter spatial risk classification.<br>• Added pointer to sensitivity analysis in main manuscript Section 4.1 (lines 423–424): *"Sensitivity analysis of input parameters are provided in Supplementary Information S4"*
2	The manuscript focuses on both PEM and alkaline electrolysers but does not distinguish their inherent differences in cooling water characteristics in the thermodynamic model and subsequent analyses. In practice, PEM electrolysers operate at lower temperatures (80–90°C) compared to alkaline electrolysers (100–150°C), leading to distinct heat dissipation profiles and cooling water requirements.	We thank the reviewer for raising this point and welcome the opportunity to clarify both the technology scope of our manuscript and the modeling rationale.<br>We would first respectfully note that, based on our survey of the commercial electrolyzer literature, commercial alkaline and PEM electrolyzers operate in largely overlapping temperature envelopes rather than the distinct ranges suggested by the comment. Independent reviews consistently place commercial alkaline electrolyzers at 60–90 °C [Buttler and Spliethoff, 2018; Shiva Kumar and Lim, 2022] and commercial PEM systems at 50–80 °C [Carmo et al., 2013; Buttler and Spliethoff, 2018]. We did not find support in the reference literature we surveyed for a distinct 100–150 °C commercial AEL operating range; if the reviewer has a specific reference in mind we would be grateful to consider it.<br>Second, regarding the modeling approach: the thermodynamic model is deliberately formulated at the cooling tower level. The driving term is the total stack heat rejection $\dot{Q}_{\mathrm{loss}}$, which is governed by hydrogen output, electrical efficiency, and the higher heating value of hydrogen (Eq. 1 of the main text). Combined with ambient psychrometric conditions, this sets the evaporative water	• Added clarifying paragraph in Section 4.1 (lines 406–415) explaining that the model is formulated at the cooling tower level and that commercial PEM (50–80 °C) and commercial AEL (60–90 °C) operate in largely overlapping temperature envelopes, making the technology-agnostic formulation a deliberate scope choice.<br>• Added three new bibliography references to support the overlapping commercial temperature envelope: Carmo et al. 2013 [ref 51], Buttler and Spliethoff 2018 [ref 52],

		demand at the tower. Technology-specific details of the upstream heat transfer chain are deliberately not resolved at this level of abstraction; these are more appropriately treated at plant-engineering scale rather than at the global climatological scale of our study. The unified formulation is consistent with the overlapping temperature envelope documented above and with the joint capacity treatment of PEM and AEL throughout the paper. We have added a clarifying paragraph to Section 4.1 (including mentioned references) of the Methods making this scope choice and the temperature overlap explicit, with the supporting references cited inline.<br>Finally, to quantify the residual effect of the small stack temperature difference that does exist within the commercial envelope, we note that it does not influence outputs heavily. The new sensitivity analysis added in response to Reviewer 1 Comment 1 (Fig. S2, tornado chart) shows that variations in approach temperature produce changes in specific cooling water consumption that are an order of magnitude smaller than those driven by electrolyzer efficiency. The qualitative conclusions of the manuscript, the spatial risk classification, the PV/wind asymmetry, and the identification of Other Solutions regions are therefore robust to the small thermal differences between commercial PEM and AEL systems.<br>We hope these clarifications, together with the added Methods paragraph, address the reviewer's concern and make the unified modeling approach more transparent to readers.	Shiva Kumar and Lim 2022 [ref 53].
3	The manuscript notes that green hydrogen projects in arid regions must adopt non-freshwater sources (e.g., seawater desalination) but only briefly mentions that desalination is	We thank the reviewer for this helpful suggestion. We agree that the discussion of alternative water-supply strategies should be supported more quantitatively. In response, we have added a new Supplementary Information section entitled “Energy and Cost Penalty of Seawater Desalination for Cooling Water Supply,” in	• Added quantitative energy and cost penalty estimate in Discussion (lines 323–329 and 337–339): incremental energy penalty

	"technically feasible but faces brine management challenges." It fails to quantify the energy, cost, and water footprint impacts of non-freshwater sources for evaporative cooling, nor does it integrate desalination/reclaimed water into the water risk index framework. This makes the recommendation of "alternative cooling strategies" lack quantitative support.	which we provide a first-order estimate of the incremental energy and cost penalty associated with supplying the additional cooling-water demand identified in this study via seawater reverse osmosis (SWRO).<br>Specifically, using the cooling-water demand range derived from our thermodynamic model (20–40 L kg^{-1} H$_2$), combined with literature-based ranges for SWRO specific energy consumption, levelized cost of water, and brine-management surcharge, we estimate an incremental desalination energy penalty of approximately 0.08–0.25 kWh kg^{-1} H$_2$, corresponding to about 0.15–0.50% of system-level electrolysis energy demand. The associated incremental cost penalty is estimated at 0.02–0.09 USD kg^{-1} H$_2$, equivalent to roughly 0.3–4.5% of current commercial LCOH estimates. We further show that, compared with reaction-water-only desalination assessments, including cooling demand increases the desalination-related energy and cost penalty by approximately a factor of three to five.<br>We have incorporated these results into the Discussion section to provide quantitative support for the statement that desalination is a plausible mitigation pathway for coastal hydrogen hubs, while also emphasizing that its direct energy and cost penalties remain modest relative to total hydrogen production costs. At the same time, we retain the important caveat that brine management can impose non-negligible environmental and operational burdens, which should be accounted for in broader techno-economic and life-cycle assessments.<br>Regarding the reviewer's suggestion to integrate desalination or reclaimed water directly into the Water Risk Index, we clarified the methodological scope of the index in Section 4.3. The RI is intentionally formulated as a baseline screening metric for	≈ 0.08–0.25 kWh kg^{-1} H_2 (0.15–0.50 % of system-level electrolysis energy) and incremental cost penalty ≈ 0.02–0.09 USD kg^{-1} H_2 (0.3–4.5 % of cost-optimized 2030 NZE LCOH)<br>• Added explicit benchmark statement that including cooling demand raises the desalination penalty by approximately a factor of three to five relative to reaction-water-only assessments<br>• Added dedicated SI Section 5 *"Energy and Cost Penalty of Seawater Desalination for Cooling Water Supply"* with detailed calculation procedure, input parameters (Table S3), worked equations (Eqs. 20–27), and cross-check against Beswick et al. and Khan et al. reaction-water-only results<br>• Added three new bibliography references: Caldera and Breyer 2017 [SI ref 18], Kumar et al.

		freshwater-based evaporative cooling, designed to identify regions where this cooling pathway would place pressure on local water resources. Alternative supply options such as desalination or reclaimed water are therefore interpreted as mitigation strategies outside the core index, rather than embedded assumptions within it. We have added a clarifying sentence to the manuscript to make this scope choice explicit.	2024 in main [ref 44], Khan et al. 2021 [SI ref 23].<br>• Added clarifying sentence to Section 4.3 (lines 465–469) explicitly stating that the Risk Index is a baseline screening metric for freshwater-based evaporative cooling, with desalination and reclaimed-water supply treated as mitigation options outside the core index rather than embedded assumptions — this directly addresses the reviewer's suggestion to integrate desalination into the RI framework
4	The model is based on long-term climatological averages (2010–2020) from ERA5 reanalysis data and does not account for the impacts of short-term climate extremes (e.g., heatwaves, droughts) on cooling water demand. In actual operation, extreme high temperatures can significantly increase evaporative cooling requirements, exacerbating local water supply-demand imbalances. Ignoring this factor leads to	We thank the reviewer for this important comment. We would like to clarify that seasonal climatic variability is an explicit part of the present analysis. In Section 2.1 and Figures 2 and 3, we quantify seasonal deviations in cooling-water demand relative to the annual climatology and show that these can reach up to approximately ±25% in many regions, with peak summer conditions pushing otherwise moderate locations into higher-demand regimes. The Discussion section also highlights the operational relevance of these seasonal peaks, including implications for cooling-system selection, seasonal water allocation, and load shifting.<br>We agree, however, that the current framework does not	• Added explicit limitations sentence in the Discussion (lines 389–392) clarifying that short-duration heatwave and drought events are not resolved by the 2010–2020 climatological framework and may temporarily increase cooling-water demand beyond the

	a gap between model results and real operational scenarios.	explicitly resolve short-duration sub-seasonal extremes such as individual heatwave or drought events. This is a consequence of the study's scope, which is to provide a global, spatially resolved climatological screening of cooling-water demand and water risk rather than a plant-level operational simulation. To make this boundary clearer, we have added a sentence in the Discussion explicitly stating that short-duration extremes are not captured by the 2010–2020 climatological averages and that such events may temporarily increase cooling-water demand above the seasonal average values reported here. We also note this as an important direction for future site-specific analysis.	reported seasonal averages.
5	Some figures have incomplete annotations (e.g., unlabeled vertical axis scales in Fig. 4, confusing subplot labels in Fig. 7). Standardize all figure formats, supplement missing scales, units, and legend explanations to meet the journal's publication requirements.	We thank the reviewer for this comment. While the original figures included axis labels, scales, and legend information, we revised the presentation of Figures 4 and 7 to improve readability and consistency. Specifically, we standardized panel labeling and typography, clarified caption wording, and made the legend and overlay categories more explicit. These changes improve interpretability without altering the underlying results.	• Figure 4 caption expanded to clarify axis labeling and red box annotation ("*Electrolysis capacity distribution by water-stress category and evaporative-cooling water demand. Cell colors indicate total electrolysis capacity [MW]...*")<br>• Figure 7 caption expanded with explicit panel-by-panel description and legend explanation ("*Panels (a) and (b) show global photovoltaic (PV) and onshore wind capacity factors, respectively. Panels (c) and (d) show*

			locations in the top quartile of PV and onshore wind capacity factors...")<br>• Figure 7 axes now include longitude/latitude tick labels (visible in updated panels)
	**Reviewer 2**	**Response**	**Changes**
6	My main concern with this manuscript is that it focuses only on evaporative cooling towers. It seems to disregard other options that address the water scarcity concerns and balances cost of cooling systems. Although it is reasonable that once through and dry cooling systems are not analysed the option of adiabatic cooling is ignored, where cooling is for the main part a dry cooler and water is only used when ambient temperature reaches a threshold and when there is a suitable difference in dry and wet bulb temperatures. This approach can lead to smaller and lower cost cooling system than dry cooling and lower water use than evaporative cooling towers and in some regions a reasonable balance between water use and cost.	We thank the reviewer for this important comment and agree that adiabatic cooling represents a relevant mitigation option in water-constrained regions. In response, we have revised the manuscript to discuss adiabatic cooling explicitly in the Discussion section. We now describe it as an intermediate pathway between evaporative and fully dry systems, in which heat is removed primarily through dry cooling while limited water use is activated only above ambient-temperature thresholds or during peak thermal conditions. This allows substantially lower aggregate water demand than conventional evaporative cooling while avoiding part of the efficiency and cost penalties associated with fully dry cooling.<br>We also broadened the interpretation of the “Other Solutions” Water Risk Index category to state explicitly that, in such regions, alternatives may include not only dry cooling and non-freshwater sourcing, but also adiabatic/hybrid cooling configurations. In addition, we added a short limitation statement clarifying that adiabatic cooling is not represented explicitly in the present global framework because its performance depends on site- and design-specific operating thresholds and transient ambient conditions,	• Added dedicated paragraph in Discussion (lines 348–359) introducing adiabatic cooling as an intermediate mitigation pathway between evaporative and dry systems, describing its operating principle (heat removed primarily through dry cooling with limited water activation above ambient-temperature thresholds), and noting its reduced aggregate water demand relative to conventional evaporative towers<br>• Added explicit reference to the ATCO ScaleH2 ARENA-supported feasibility study

		which are difficult to parameterize in a globally harmonized manner.	[new ref 28] as a concrete industry example of adiabatic cooling being selected for water-constrained sites<br>• Added sentence in Introduction (lines 66–71) acknowledging that Australian feasibility studies already report consideration of water-saving cooling configurations including adiabatic cooling<br>• Added adiabatic cooling as a named limitation and future-work direction in the limitations paragraph (lines 389–396), noting that its site- and design-specific operating thresholds do not transfer readily to a globally harmonized screening model<br>• Updated Section 4.3 (lines 461–463) to explicitly list adiabatic/hybrid cooling alongside dry cooling and desalination as mitigation

			options for Other Solutions zones
7	Whereas industry appears to already understand the issue of high-water demand in evaporative cooling towers and its impact in areas where water is scarce. In Australia multiple industry projects have reported that they select adiabatic cooling that has a much lower aggregate water demand. The available industry reports in Australia from ARENA knowledge sharing site (1) are of industry projects after the cited ARUP 2022 report in this manuscript (Reference 3, that appears to also be reference 15) that also ignores the adiabatic cooling option. As such I question whether this manuscript is analysing an industry problem that has already been solved.<br>I recommend this manuscript is revised to also include adiabatic cooling that appears to be what industry is considering for water scarce countries.	We appreciate this perspective and agree that some project developers have already begun to respond to water constraints through alternative cooling choices. We have therefore added an explicit discussion of recent Australian feasibility studies as practical examples of this emerging design response. For example, the ARENA-supported ATCO ScaleH2 feasibility study identifies adiabatic cooling as the preferred cooling option for the assessed site conditions and describes its operation as a primarily dry system with water use activated above a temperature threshold. The report also notes that this configuration can reduce average water use relative to conventional evaporative cooling, although with more peaked water demand during hotter periods.<br>At the same time, we respectfully suggest that such project-level choices do not make the present analysis redundant. Rather, they reinforce the central finding of the manuscript: namely, that cooling-water constraints are real, spatially heterogeneous, and consequential enough to influence technology selection in practice. The contribution of the present study is not to claim that evaporative cooling will be used everywhere, but to provide a globally spatial, seasonally resolved screening framework that identifies where freshwater-based evaporative cooling is likely to become problematic and where alternative solutions are likely to be required. In that sense, the industry examples cited by the reviewer are consistent with, and indeed support, the practical relevance of the risk patterns quantified here.<br>Accordingly, instead of expanding the model to a full global representation of adiabatic cooling, we revised the manuscript to position adiabatic cooling more clearly as one of the relevant mitigation pathways indicated by high-risk regions in our	• Added sentence in the Introduction (lines 66–71) noting that recent feasibility studies in water-constrained regions such as Australia already consider water-saving cooling options, including adiabatic cooling, but that such project-level decisions remain case-specific and lack a systematic global quantitative basis.<br>• Added discussion of the ARENA-supported ATCO ScaleH2 example in the Discussion (lines 347–359) as practical evidence that regional water-risk patterns already influence cooling-technology selection in industry.

		framework, alongside dry cooling, desalination-coupled cooling, and reclaimed-water supply.	

<u>For completeness, the following references have been newly added in this revision:</u>

Ref #	Citation	Purpose
[28]	ATCO Australia, ScaleH2 Feasibility Study Final Feasibility Summary Report, ARENA, July 2025	R2C6/R2C7 — industry evidence on adiabatic cooling
[51]	Carmo, Fritz, Mergel, Stolten 2013, *Int. J. Hydrogen Energy* 38(12):4901–4934<br>Note	R1C2 — commercial PEM operating temperature
[52]	Buttler and Spliethoff 2018, *Renewable and Sustainable Energy Reviews* 82:2440–2454	R1C2, R1C3 — temperature envelopes and system-level electrolysis energy
[53]	Shiva Kumar and Lim 2022, *Energy Reports* 8:13793–13813	R1C2 — commercial AEL operating temperature
SI [18]	Caldera and Breyer 2017, *Water Resources Research* 53(12):10523–10538	R1C3 — SWRO LCOW and SEC
SI [23]	Khan et al. 2021, *Energy Environ. Sci.* 14:4831–4839	R1C3 — reaction-water-only benchmark
[48]	Khan et al. 2021, *Energy Environ. Sci.* 14:4831–4839	R1C3 — reaction-water-only benchmark
